# The extrafollicular response is sufficient to drive initiation of autoimmunity and early disease hallmarks of lupus

**DOI:** 10.3389/fimmu.2022.1021370

**Published:** 2022-12-14

**Authors:** Lasse F. Voss, Amanda J. Howarth, Thomas R. Wittenborn, Sandra Hummelgaard, Kristian Juul-Madsen, Kristian S. Kastberg, Mathias K. Pedersen, Lisbeth Jensen, Anastasios D. Papanastasiou, Thomas Vorup-Jensen, Kathrin Weyer, Søren E. Degn

**Affiliations:** ^1^ Department of Biomedicine, Aarhus University, Aarhus, Denmark; ^2^ Department of Health Technology, Technical University of Denmark, Kongens Lyngby, Denmark; ^3^ Department of Biomedical Sciences, University of West Attica, Athens, Greece

**Keywords:** autoimmunity, B cells, germinal centers, extrafollicular responses, autoantibodies, systemic lupus erythematosus, TLR7

## Abstract

**Introduction:**

Many autoimmune diseases are characterized by germinal center (GC)-derived, affinity-matured, class-switched autoantibodies, and strategies to block GC formation and progression are currently being explored clinically. However, extrafollicular responses can also play a role. The aim of this study was to investigate the contribution of the extrafollicular pathway to autoimmune disease development.

**Methods:**

We blocked the GC pathway by knocking out the transcription factor Bcl-6 in GC B cells, leaving the extrafollicular pathway intact. We tested the impact of this intervention in two murine models of systemic lupus erythematosus (SLE): a pharmacological model based on chronic epicutaneous application of the Toll-like receptor (TLR)-7 agonist Resiquimod (R848), and 564Igi autoreactive B cell receptor knock-in mice. The B cell intrinsic effects were further investigated *in vitro* and in autoreactive mixed bone marrow chimeras.

**Results:**

GC block failed to curb autoimmune progression in the R848 model based on anti-dsDNA and plasma cell output, superoligomeric DNA complexes, and immune complex deposition in glomeruli. The 564Igi model confirmed this based on anti-dsDNA and plasma cell output. *In vitro*, loss of Bcl-6 prevented GC B cell expansion and accelerated plasma cell differentiation. In a competitive scenario *in vivo*, B cells harboring the genetic GC block contributed disproportionately to the plasma cell output.

**Discussion:**

We identified the extrafollicular pathway as a key contributor to autoimmune progression. We propose that therapeutic targeting of low quality and poorly controlled extrafollicular responses could be a desirable strategy to curb autoreactivity, as it would leave intact the more stringently controlled and high-quality GC responses providing durable protection against infection.

## Highlights:

-A genetic GC block fails to prevent autoimmune progression in two lupus models
-An intrinsic GC block drives B cell differentiation into terminally differentiated plasma cells *in vitro*
-B cells harboring a GC block competitively contribute to the plasma cell compartment in an autoreactive setting *in vivo*
-Lupus mice with a GC block display immune complex deposition in kidney glomeruli that is indistinguishable from their wild-type counterparts

## Introduction

Many autoimmune diseases, such as systemic lupus erythematosus (SLE) and Sjögren’s syndrome, are characterized by the development of autoantibodies targeting nuclear antigens (1, 2). Such antibodies can be produced by B cells *via* the extrafollicular pathway and the germinal center (GC) pathway (3). The extrafollicular pathway leads to the rapid expansion and differentiation of B cells to plasmablasts (PBs) and short-lived plasma cells (PCs), providing early protection against pathogens. The GC pathway is slower, but enables a higher-quality response characterized by extensive somatic hypermutation and affinity maturation, robust memory cell generation, and production of long-lived PCs (4). At an individual B cell level, the initial affinity for antigen governs the decision between extrafollicular and GC differentiation (5). While both pathways can support class-switch recombination and affinity maturation, the antibody class diversity and extent of somatic hypermutation is much greater through the GC pathway (6, 7).

Upon initial activation of B cells by their cognate antigen, they can either T-independently or T-dependently form an extrafollicular focus. Here they proliferate, as well as potentially class-switch, and may additionally undergo a low degree of somatic hypermutation, before they differentiate into PCs. In the context of autoimmune diseases, initial B cell reactivities often target autoantigenic components that carry endogenous TLR ligands capable of stimulating them independently of T cells (8–10). These seem strictly limited to components topologically linked to the B cell receptor (11). Although TLR9 was originally reported to be a main driver of autoreactive B cell activation (10), more recent insights suggest RNA recognition through TLR7, and potentially TLR3, is the most important contributor to break-of-tolerance in B cells (9, 12, 13), with TLR9 playing a counterbalancing role (14–16). Extrafollicular responses have been noted as a prevalent phenomenon both in mouse models of autoimmunity (17, 18) and lupus patients (19). Interestingly, the signals that drive initial B cell activation also seem to limit the extent of affinity maturation (20), perhaps by disproportionately favoring the extrafollicular differentiation path. However, as autoimmune disease progresses, the autoantigenic response often broadens, leading to inclusion of more heavily T-dependent reactivities (21). This broadening of the response, which is termed epitope spreading, has been observed even before the onset of clinical symptoms (22, 23).

Epitope spreading is thought to occur through the GC pathway. In this alternate outcome of initial B cell activation, the B cells may form a primary focus in the interfollicular region, where they undergo limited proliferation and may class-switch (24, 25). They then subsequently co-migrate with cognate T cells into the follicle, where they can form a GC. In the GC, the B cells proliferate rapidly and form a ‘dark zone’, and are now termed centroblasts (4). The centroblasts undergo somatic hypermutation to diversify their B cell receptors and subsequently migrate to the ‘light zone’, where they scan follicular dendritic cells for antigen. The B cells that display the highest affinity for antigen can competitively acquire antigen and present derived peptides to T follicular helper (T_FH_) cells. B cells that receive cognate T cell help may return to the dark zone for another round of division and hypermutation. B cells that do not receive help perish through programmed cell death and are engulfed by tingible body macrophages (4). Due to the power of the GC pathway, and the risk for inadvertent emergence of novel (auto)reactivities that are distinct from the original antigenic target, it is subject to stringent control. The requirement for T cell selection subsequent to every successive round of hypermutation, a phenomenon termed linked recognition, restricts inadvertent broadening of the response. An additional layer of control appears to be exerted by a specialized subset of T regulatory cells, termed T follicular regulatory (T_FR_) cells (26). Nonetheless, these mechanisms appear to fail in autoimmune disease, which frequently display rampant GC activity (27, 28).

It was previously found that a genetic GC block prevented development of collagen-induced arthritis (29). Hence, we hypothesized that the GC pathway is critical to the autoimmune process, and that blocking the GC pathway would prevent development of SLE. Surprisingly, a global block in the GC pathway *in vivo* in two independent SLE models did not mitigate autoimmune disease. In an *in vitro* GC B cell culture system, GC blocked B cells expanded to a lesser extent, but were found to more rapidly develop into PBs and PCs. In a competitive scenario *in vivo*, GC blocked B cells competed efficiently with their wild-type counterparts, disproportionate to their inability to participate in GCs. Our determination of the relative contributions of the extrafollicular and GC pathways to autoimmune progression highlights a critical role of extrafollicular responses in driving autoimmune development.

## Materials and methods

### Mice

The Bcl-6^flx/flx^ strain (30) and congenic B6.CD45.1 (B6.SJL-Ptprc^a^ Pepc^b^/BoyJ) were purchased from Jackson Laboratories (stock no. 023737 and 002014, respectively). Aicda-Cre transgenic mice (31) were kindly provided by Meinrad Busslinger. Aicda-Cre and Bcl-6^flx/flx^ strains were intercrossed to generate Aicda-Cre+ and Aicda-Cre- Bcl-6^flx/flx^ littermates. 564Igi mice (13) (B6.Cg-Igh^tm1(Igh564)Tik^Igk^tm1(Igk564)Tik^/J) were kindly made available by Theresa Imanishi-Kari. The 564Igi line was crossed to Aicda-Cre Bcl6^flx/flx^ to generate 564Igi H^+/+^K^+/+^ Aicda-Cre+/- Bcl6^flx/flx^ littermates. Mice were housed in the Animal Facility at our institution, under SPF conditions, on a 12-hour light/dark cycle with standard chow and water *ad libitum*. Both male and female mice were used (**Supplementary Tables 1**, **2**), aged 8-14 weeks at initiation of experiments. See also section on ARRIVE reporting and statistical analyses.

### R848 treatment protocol

Mice were briefly anesthetized in continuous flow of 4% isoflurane, then treated topically on the right ear with 1 mg R848/mL acetone or did not receive any treatment (Untreated/Unt). Treatment was performed using a 15 cm, latex- and PVC-free cotton-tipped applicator, which was dipped into the R848 solution until soaked, then rolled on the inner leaflet of the ear, and this process was repeated with application on the outer leaflet of the ear. This treatment procedure was carried out three times/week for 4 weeks for each mouse.

### Mixed bone marrow chimeras

Mixed bone marrow chimeras were set up as previously described (32). Recipient mice were irradiated with 9 Gy in a MultiRad 350 (Faxitron), with 350 kV, 11.4 mA, a Thoraeus filter [0.75 mm Tin (Sn), 0.25 mm Copper (Cu), and 1.5 mm Aluminium (Al)], and with a beam-distance of 37 cm. Irradiated recipients were kept on antibiotic water (either 1 mg sulfadiazine together with 0.2 mg trimethoprim per mL drinking water, or 0.25 mg amoxicillin per mL drinking water) to avoid any opportunistic infections. On the following day, donor mice were anesthetized with 4% isoflurane and euthanized. Femora, fibulae/tibiae, ossa coxae and humeri were harvested, mechanically cleaned and rinsed in FACS buffer. The bone marrow (BM) cells were released from the harvested bones by crushing and the cell extract was then passed through a 70 µm cell strainer. The donor BM cells were then counted in a Cellometer K2 cell counter (Nexcelom). Cells were pelleted by centrifugation (200 g, 10 min, 4°C) and resuspended to 1*10^8^ cells/mL. Donor cells from three different mice were then mixed according to the proportions mentioned in the figure legend. The donor cell mixtures were used to reconstitute the recipient mice by retroorbital injection of 200 µL (containing a total of 20*10^6^ cells) into each recipient mouse. The reconstituted recipient mice were placed on antibiotic water the following 14 days.

### Tissue preparation

Mice were anesthetized with isoflurane (055226, ScanVet), blood samples from the retroorbital plexus were collected, and mice were euthanized using 100-150 mg/kg sodium pentobarbital (450009, Dechra Veterinary Products). Mesenteric lymph nodes (MesLN) and inguinal lymph nodes (IngLN) were removed, the splenic artery was clamped with a hemostat, and the spleen was removed. The mice were perfused intracardially with PBS (BE17-515Q, Lonza) to remove the blood, and subsequently perfused with 4% w/v paraformaldehyde (PFA) (1.04005.100, Merck) in PBS to fix the tissues. Finally, kidneys and auricular lymph nodes (AurLNs) were removed.

Collected blood samples were centrifuged at 3,000 *g* for 10 minutes, the supernatant was collected, and centrifuged again at 20,000 *g* for 3 minutes. Serum samples were stored at -20°C. The spleen and AurLNs were directly embedded in Tissue-Tek O.C.T. media (4583, Sakura Finetek) and frozen at -20°C for histology. The kidneys were kept in 4% w/v PFA for 24 hours, and then changed to 30% w/v sucrose in PBS. A small part of the spleen as well as IngLN and MesLN were stored in fluorescence-activated cell sorting (FACS) buffer [PBS, 2% heat-inactivated fetal calf serum (FCS), 1 mM ethylenediaminetetraacetic acid (EDTA)] for FACS typing.

### Flow cytometry

Flow cytometry was performed according to standard procedure. Spleen, IngLN and MesLN were harvested, stored into ice-cold FACS buffer, and mechanically dissociated using pestles. Spleen and LNs were filtered through 70 µm cell strainers. Spleen samples were centrifuged at 200 g for 5 minutes at 4°C, lysed in RBC lysis buffer (155 mM NH_4_Cl, 12 mM NaHCO_3_, 0.1 mM EDTA), incubated at RT for 3 minutes, centrifuged, and finally resuspended in FACS buffer or calcium-containing buffer (PBS, 20 mM HEPES, 145 mM NaCl, 5 mM CaCl_2_, 2% FBS) when Annexin-V was included in the panels. Samples were filtered through 70 µm cell strainers. Twenty µL Fc-block (553142, BD) diluted 1:50 in PBS and 100 µL of each sample was added onto a 96-well plate and incubated for 5-10 minutes. Antibodies and fixable viability dye (65-0865-14, ThermoFisher Scientific) were diluted in FACS buffer or calcium-containing buffer as indicated below. 100 µL antibody mix was added to each sample well and incubated for 30 minutes on ice. The plate was centrifuged at 200 g for 5 minutes, supernatant was removed, and cells were fixed for 30 minutes in PBS, 0.9% formaldehyde (F1635, Sigma-Aldrich) at RT. Later, the plates were centrifuged at 200 g for 5 minutes, the supernatant discarded, and the samples resuspended in FACS buffer or calcium-containing buffer. Flow cytometry evaluation was performed the following day using a 4-laser (405 nm, 488 nm, 561 nm, 640 nm) LSRFortessa analyzer (BD instruments). The following antibodies and reagents were used for flow cytometry experiments: Annexin-V-AF488 (A13201, ThermoFisher Scientific, 1:500), B220-PB clone RA3-6B2 (558108, BD, 1:500), B220-PerCP-Cy5.5 clone RA3-6B2 (561101, BD Biosciences, 1:500), CD4-PerCP clone RM4-5 (100538, BioLegend, 1:500), CD8-PerCP-Cy5.5 clone SK1 (565310, BD, 1:500), CD38-PE-Cy7 clone 90 (102718, BioLegend, 1:500), CD45.1-FITC clone A20 (110706, BioLegend, 1:500), CD45.2-APC clone 104 (109814, BioLegend, 1:500), CD95-PE clone Jo2 (554258, BD, 1:500), CD138-BV650 clone 281-2 (564068, BD, 1:500), 9D11-biotin (hybridoma kindly provided by Elisabeth Alicot, Boston Children’s Hospital, produced, purified and biotinylated in-house, 1:300), Ly6G/C-APC-R700 clone RB6-8C5 (565510, BD, 1:500), Viability Dye eFlour 780 (65-0865-14, Thermo Fisher Scientific, 1:2000), Streptavidin-BV786 (563858, BD Biosciences, 1:500), hCD2-PB clone RPA-2.10 (300236, BioLegend, 1:200), IgD-AF488 clone 11-26c.2a (405718, BioLegend, 1:500), IgMb-BV510 clone AF6-78 (742344, BD OptiBuild, 1:500), TACI-AF647 clone 8F10 (558453, BD Biosciences, 1:500), CD45.2-BV786 clone 104 (563686, BD Horizon, 1:500), CD19-AF700 clone 1D3 (557958, BD Pharmingen, 1:500).

### Quantum dot coupling of antibody

Quantum Dot (QD) antibody coupling was done using SiteClick Qdot 655 Antibody Labeling Kit (Molecular Probes, S10453) according to manufactures instructions. In brief, antibody [either “14D12” rat IgG2a to mouse MBL-C (Hycult Biotech), or “RTK2758” rat IgG2a isotype control (Abcam)] was concentrated in antibody preparation buffer to a concentration of 2 mg/mL or above. Next, carbohydrates on the antibody were modified by the incubation with β-galactosidase for 4 h at 37°C. Azide modification was achieved through incubation with uridine diphosphate glucose-GalT enzyme overnight at 30°C. Antibody with modified carbohydrates was purified and concentrated through a series of centrifugation steps using a molecular-weight cutoff membrane concentrator, and the buffer was simultaneously changed to 20 mM Tris, pH 7.0. Finally, 5′-dibenzocyclooctyne-modified QD nanocrystals were coupled overnight at 25°C and stored at 4°C until further use.

### Nanoparticle tracking analysis

Samples for Nanoparticle Tracking Analysis (NTA) were analyzed using a NanoSight NS300 system (Malvern Panalytical) as previously described (33). The system was configured with a 405 nm laser, a high-sensitivity scientific complementary metal–oxide–semiconductor Orca Flash 2.8/Hamamatsu C11440 camera (Malvern Panalytical), a syringe pump, and for fluorescence measurements, a 650 nm long-pass filter was used. The sample chamber was washed twice with 1 mL PBS containing 1 mM EDTA (PBS/EDTA) before each measurement. All samples were thoroughly mixed before measurement and were injected into the sample chamber using 1-mL syringes. The measurement script comprised temperature control at 23°C, followed by a 20 s flush at a flowrate mark 1000. Next, sample advancement was stabilized by a 120 s advancement at flowrate mark 10. Recordings were captured continuously during a steady flow at flowrate mark 10 with five 60-s recordings separated by 5-s lag time between each sample. The videos were collected and analyzed using NanoSight software (version 3.4 with a concentration upgrade; Malvern). Automatic settings were used for the minimal expected particle size, minimum track length, and blur setting. Camera sensitivity and detection threshold were adjusted according to sample composition and kept constant for all samples to be directly compared. For fluorescence mode, the camera level was set to maximum (mark 16), and the detection threshold was set to minimum (mark 2). Serum samples from mice were analyzed in a 1:20 dilution in PBS/EDTA with a 1:20,000 dilution of MBL-C–specific (14D12) or isotype Antibody-QD reporters. A 50 nm cutoff was established for all samples to exclude unbound QD conjugates as well as QD conjugates bound to smaller forms of MBL-C.

### Immunohistochemical labelling of kidney tissue

Immunohistochemical labelling of kidney tissue was performed according to standard protocol. After perfusion fixation, kidneys were stored in PBS with 30% sucrose and 0.1% sodium azide. For paraffin-embedding, the kidneys were washed in 10 mM PBS several times, dehydrated in 70%, 96%, and 99% ethanol for 2 hours, respectively, before they were transferred to xylene overnight. The day after, kidneys were embedded in paraffin. Paraffin embedded kidney sections (2 µm) were cut on a Leica RM 2165 microtome (Leica, Wetzlar, Germany) and dried at 60°C for 1 h. For immunofluorescence (IF) labelling, sections were placed in xylene overnight, rehydrated in graded alcohols, and heated in TEG buffer (10 mM Tris, 0.5 mM EGTA buffer, pH 9) at ~100°C for 10 min to induce epitope retrieval. Sections were subsequently cooled for 30 min, incubated in 50 mM NH_4_Cl in 0.01 mM PBS for 30 min, and incubated at 4°C with primary antibody overnight. The following day, sections were washed, incubated with secondary antibody for 1 h, and coverslips were mounted using mounting medium (Dako fluorescence Mounting medium, #S3023). Immunofluorescence images were acquired by a confocal laser-scanning microscope (LSM 800 with Airyscan, Carl Zeiss Gmbh, Jena, Germany) and processed using ZEN lite 3.4 (Blue edition). For immunoperoxidase (IP) labelling, sections were prepared as stated above. In addition, endogenous peroxidase was blocked by placing sections in 30% H_2_O_2_ in methanol for 30 min and peroxidase-conjugated secondary antibodies were used. Furthermore, the sections were incubated with DAB, counterstained with Mayer’s haematoxylin (Sigma-Aldrich), dehydrated in graded alcohols, cleared in xylene and mounted with coverslips using Eukitt (Sigma-Aldrich). For periodic acid-Schiff (PAS) stainings, sections were incubated in 1% periodic acid for 10 min and treated with Schiff’s reagent for 20 min. Subsequently, all sections were counterstained with Mayer’s hematoxylin for 5 min and mounted with coverslips using Eukitt. Peroxidase-labelled and PAS-stained images were collected by a Leica DFC320 camera (Leica, Wetzlar, Germany). H&E and PAS stained kidney sections were scored by a histopathologist, blinded to the identity of the samples. High-resolution images of kidney and spleen sections were obtained using a Zeiss LSM 800 Airyscan confocal microscope with 4 lasers (405 nm, 488 nm, 561 nm, 640 nm). ZEN software was used for quantification of immunofluorescence kidney stainings. Channel intensities were adjusted for visual clarity in represented micrographs, but quantification was performed on raw images throughout. The following primary antibodies were used: anti-C3 [ab11887, Abcam, 1:25 (IP), 1:50 (IF)], anti-IgG2c [1079-08, SouthernBiotech, 1:25 (IF), 1:50 (IP)], anti-Nephrin (GP-N2, Progen, 1:100), anti-Ig [1010-08, SouthernBiotech, 1:100 (IF), 1:200 (IP)]. The following secondary antibodies were used: Donkey-a-rabbit AF488 (A21206, ThermoFisher, 1:300), Streptavidin AF647 (405237, BioLegend, 1:500), Goat-a-Guinea Pig AF488 (A11073, ThermoFisher, 1:300), Goat-a-rabbit HRP (P0448, Dako, 1:200), Streptavidin HRP (P0397, Dako, 1:200).

### Time-resolved immunofluorometric analysis anti-dsDNA measurements

A FluoroNunc Maxisorp 96-well plate was coated with 100 µg/mL salmon sperm dsDNA (AM9680, Invitrogen) in PBS and incubated overnight at 4°C. Wells were blocked with 200 µL TBS containing 1% bovine serum albumin (BSA) (A4503, Sigma-Aldrich) for 1 hour at RT and washed 3 times with TBS/Tw [TBS containing 0.05% v/v Tween-20 (8.17072.1000, Merck)]. Samples, standards and quality controls were diluted in TBS/Tw containing 5 mM EDTA and 0.1% w/v BSA, and were subsequently loaded onto the plate in duplicates. The plate incubated at 37°C for 1 hour. Then, wells were washed 3 times in TBS/Tw and incubated with biotinylated antibody (Table 2.5) at 37°C for 1 hour. Wells were washed 3 times in TBS/Tw, and Eu^3+^-tagged streptavidin (1244-360, PerkinElmer) diluted 1:1,000 in TBS/Tw containing 25 µM EDTA were subsequently added to the wells and incubated at RT for 1 hour. Finally, the wells were washed 3 times in TBS/Tw, 200 µL enhancement buffer (AMPQ99800, Amplicon) was added. The plate was shaken for 5 minutes and counts were read by a time-resolved fluorometry plate reader Victor X5 (Perkin Elmer).

### TRIMA Ig measurements

A FluoroNunc Maxisorp 96-well plate was coated with 1 µg/mL goat anti-mouse Ig in PBS and incubated overnight at 4°C. Wells were blocked in 1 mg HSA/mL TBS for 1 hour at RT and washed 3 times with TBS/Tw. Samples, standards and quality controls, diluted in TBS/Tw containing 100 µg/mL heat-aggregated human Ig, were subsequently loaded onto the plate in duplicates, and incubated overnight at 4°C. The wells were washed 3 times with TBS/Tw, and 1 µg/mL biotinylated goat anti-mouse Ig was added to the wells and incubated for 2 hours at RT. Wells were washed 3 times in TBS/Tw, and Eu^3+^-tagged streptavidin (1244-360, PerkinElmer) diluted 1:1,000 in TBS/Tw containing 25 µM EDTA were subsequently added to the wells and incubated at RT for 1 hour. Finally, the wells were washed 3 times in TBS/Tw, 200 µL enhancement buffer (AMPQ99800, Amplicon) was added. The plate was shaken for 5 minutes and counts were read by a Victor X5 time-resolved fluorometry plate reader (Perkin Elmer).

### Immunofluorescence staining of spleens and auricular lymph nodes

A Cryostar NX70 Cryostat (ThermoFisher) was used to cut 16 µm thick spleen sections or 20 µm thick auricular lymph node sections which were mounted on SuperFrost+ glass slides (Fisher Scientific). Spleen sections were either acetone or PFA fixed, auricular lymph nodes were PFA fixed. For acetone fixation, the spleen samples were rinsed in PBS and fixed in acetone for 10 minutes at room temperature (RT), whereafter the slides were rehydrated in PBS for 3 minutes. For PFA fixation protocols, the slides were washed in PBS, fixed with 4% w/v PFA for 30 min at RT, incubated in TBS (10 mM Tris, 140 mM NaCl, pH 7.4) for 30 min at RT, rinsed briefly with PBS, and incubated with permeabilization buffer (PBS, containing 2% v/v FBS, 0.1% w/v sodium azide, 0.1% v/v Triton-X100) for 45 minutes at RT. Antibodies were diluted in staining buffer (PBS, 2% v/v FBS, 0.1% w/v sodium azide). The antibody mix was centrifuged at 10,000 *g* for 5 minutes and added onto the spleen samples, where it incubated overnight at 4°C. The slides were washed once with staining buffer for 5 minutes and washed 3 times in PBS with 0.01% v/v Tween-20 for 5 minutes. Slides were spot-dried and mounted using Fluorescence Mounting Medium (S3023, Dako). Imaging for quantification of GC formation was performed using an Olympus VS120 Upright Widefield fluorescence slide scanner equipped with a digital monochrome camera (Hamatsu ORCA Flash4.0V2) and a 2/3” CCD camera, as well as single-band exciters and a filter wheel with single-band emitters (Hoechst, FITC, Cy3, Cy5, and Cy7). Fiji v. 2.1.0/1.53c was used for image processing. Channel intensities were adjusted for visual clarity in represented micrographs, but quantification was performed on raw images throughout. The following antibodies were used: CD45.1-FITC clone A20 (110706, BioLegend, 1:300), CD45.2-APC clone 104 (109814, BioLegend, 1:300), CD138-PE clone 281-2 (142504, BioLegend, 1:500), CD169-PE clone 3D6.112 (142404, BioLegend, 1:500), IgD-AF488 clone 11-26c.2a (405718, BioLegend, 1:500), Ki67-eflour660 clone SolA15 (50-5698-82, Thermo Fisher Scientific, 1:500), CD21/35-PB clone 7E9 (123414, BioLegend, 1:500).

### Purification of B cells

The spleen was harvested and placed in MACS buffer (PBS, 2% FBS, 2 mM EDTA), then it was mechanically dissociated and cells collected by filtering through 70 µm cell strainers, whereafter the cell suspension was topped up with MACS buffer until 25 mL and filtered through 70 µm cell strainers again. The cell suspension was centrifuged at 200 g for 10 minutes at 4°C, resuspended in 5 mL RBC lysis buffer (155 mM NH_4_Cl, 12 mM NaHCO_3_, 0.1 mM EDTA), and incubated for 3 minutes at RT. The reaction was stopped by adding 47 mL MACS buffer, and cells were collected by centrifugation at 200 g for 5 minutes at 4°C. The supernatant was discarded and the pellet resuspended in 3 mL MACS buffer. B cell purification kit (Miltenyi Biotec, 130-090-862) was subsequently used, according to the manufacturer’s protocol.

### iGB cultures

NB21 feeder cells, kindly provided by Garnett Kelsoe (34), were seeded into 6-well plates at a density of 520 cells/cm^2^. The following day (day 0), purified B cells were resuspended in B cell medium (BCM) (RPMI-1640 supplemented with 10% FCS, 55 µM 2-ME, 1% Pen/Strep, 1% MEM NEAA, 10 mM HEPES, 1 mM Sodium Pyruvate). B cells were pre-diluted in BCM with IL-4 (PeproTech, 214-14). From day 2 to 8, 2/3 of the total volume of BCM was collected and fresh BCM was added to reach the same final volume. 1 mL of medium from each well of the 6-well plates was collected on the final day (day 10) for TRIFMA analyses. Cells were analyzed using flow cytometry, as described above. For proliferation assay, freshly thawed Cre+ and Cre- Bcl6^flx/flx^ B cells cells were pelleted and resuspended in Tag-it Violet (BioLegend, 425101) working solution (5 µM Tag-it Violet in PBS, pH 7.4) at 10^7^ cells/mL, incubated 20 minutes at room temperature protected from light, then added 1.5 mL BCM, and centrifuged 200 g, 5 min. The supernatant was discarded and the pellet was resuspended in pre-warmed BCM, then incubated 10 minutes at 37°C. Labelled cells were seeded at either 50,000 cells per well (conditioned medium), or 5,000 cells/well (feeder cells + IL-4), in parallel with unlabeled cells at 5,000 cells/well (feeder cells + IL-4).

### ARRIVE reporting and statistical analyses

**Figure 1:**
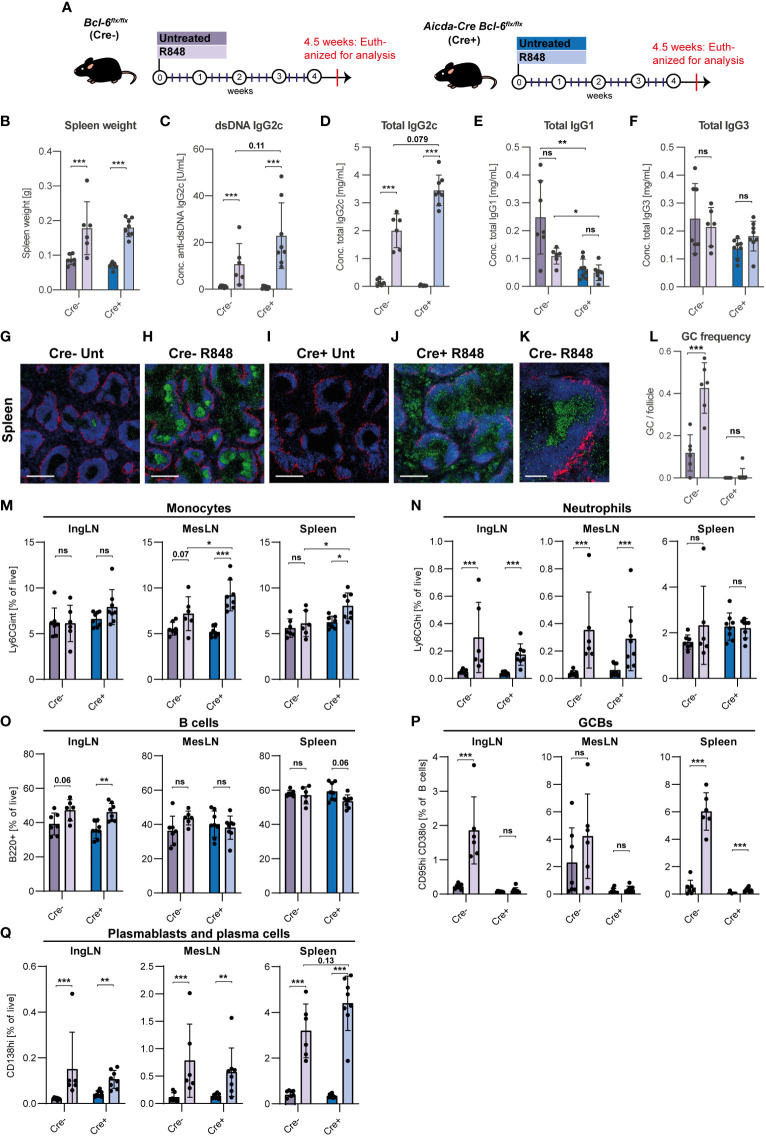
GC block fails to prevent development of autoantibodies and PB/PC expansion in lupus mice. **(A)** Overview of experimental setup. Bcl-6^flx/flx^ (Cre-, purple) and Aicda-Cre Bcl-6^flx/flx^ (Cre+, blue) mice were left untreated (dark color, n=7 and n=8, respectively) or treated with R848 (light color, n=6 and n=8, respectively). **(B)** Spleen weights. **(C)** Anti-dsDNA IgG2c. **(D)** Total IgG2c. **(E)** Total IgG1. **(F)** Total IgG3. **(G)** Representative confocal micrograph of spleen from Cre- untreated animal, stained for CD169 (red), IgD (blue) and Ki67 (green). Scale bar is 400 µm. **(H)** As G, but for Cre- treated animal. **(I)** As G, but for Cre+ untreated animal. **(J)** As G, but for Cre+ treated animal. **(K)** High-resolution image of GC from Cre- treated mouse. Scale bar=100 µm. **(L)** GC/follicle in spleen. **(M)** Flow cytometry analyses of monocyte frequencies (Ly6CG^int^) in IngLN, MesLN, and spleen. **(N)** As M, but neutrophil frequencies (Ly6CG^hi^). **(O)** As M, but B cell frequencies (B220^+^CD4^-^CD8^-^). **(P)** As M, but GCB frequencies (CD95^hi^CD38^lo^). **(Q)** As M, but PB and PC frequencies (CD138^hi^). Data pooled from two independent experiments. Bar graphs show mean ± SD. ns = p≥0.05, *p < 0.05, **p < 0.01, ***p < 0.001, based on two-way ANOVA with Holm-Sidak’s post-test.

**Figure 2:**
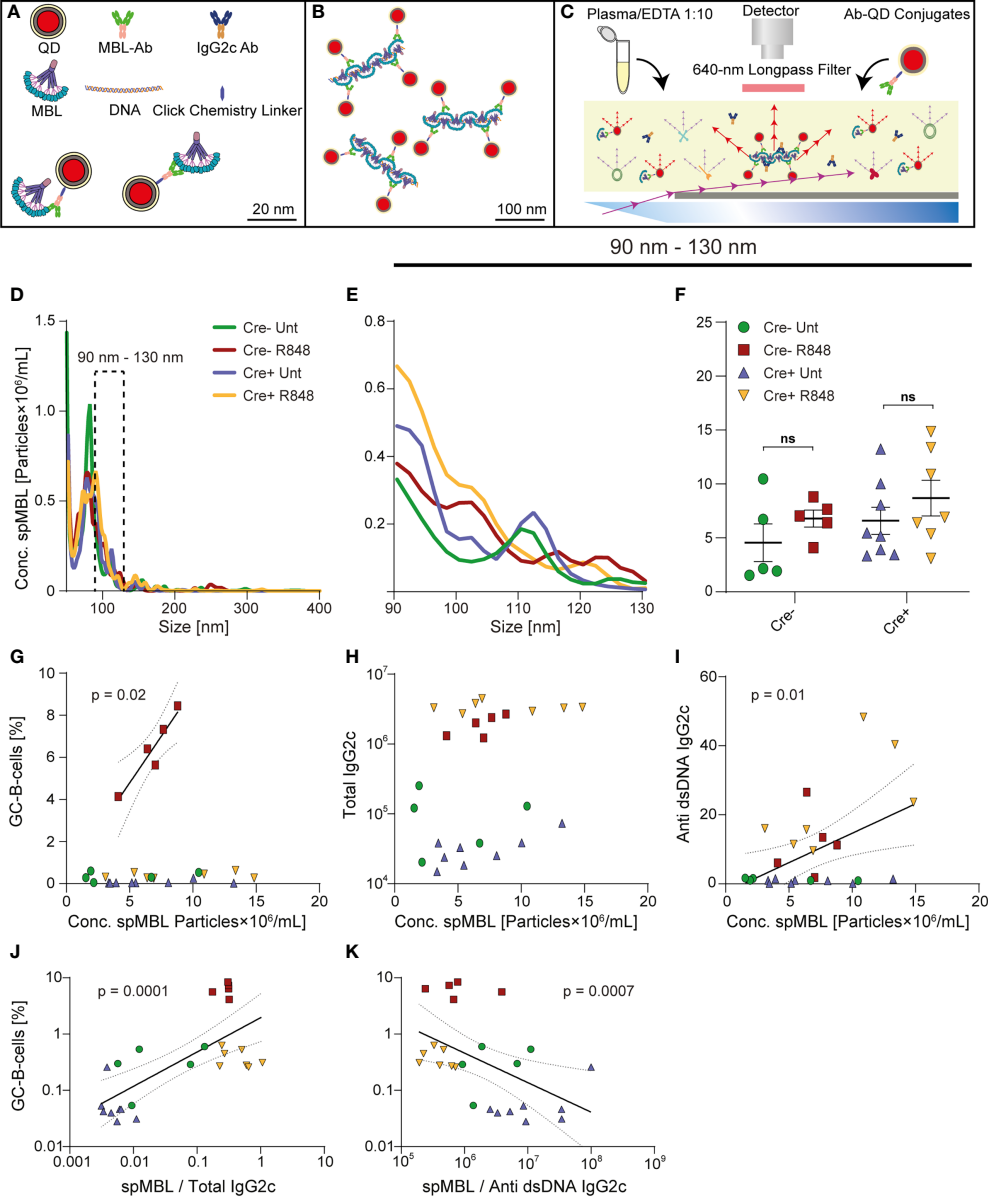
Trend towards increased levels of spMBL particles in serum from Cre+ R848 treated mice, and GC and autoantibody correlations with spMBL. **(A–C)** Schematic overview of experimental setup for spMBL analysis of serum samples. **(D)** Samples were tested for the size interval 90-130 nm from Aicda-Cre Bcl-6^flx/flx^ (Untreated: blue, R848-treated: yellow) and Bcl-6^flx/flx^ littermate controls (Untreated: green, R848-treated: red). **(E)** Zoom onto the range of 90 to 130 nm. **(F)** Graph showing mean ± SEM and individual measurements across treatment protocols for Cre- untreated (n=5), Cre- R848-treated (n=5), Cre+ untreated (n=8), and Cre+ R848-treated (n=7) mice. **(G)** Correlation analysis of GCB vs. Conc. spMBL. **(H)** Correlation analysis of total IgG2c levels vs. Conc. spMBL. **(I)** Correlation analysis of anti-dsDNA IgG2c vs. Conc. spMBL. **(J)** Correlation analysis of GCB cells vs. spMBL/total IgG2c **(K)** Correlation analysis of GCB vs. spMBL/anti-dsDNA IgG2c. Two-way ANOVA with Holm-Sidak’s *post hoc* test was used to analyze the data in F. Linear regression models were used to analyze G-K. ns = p≥0.05.

**Figure 3:**
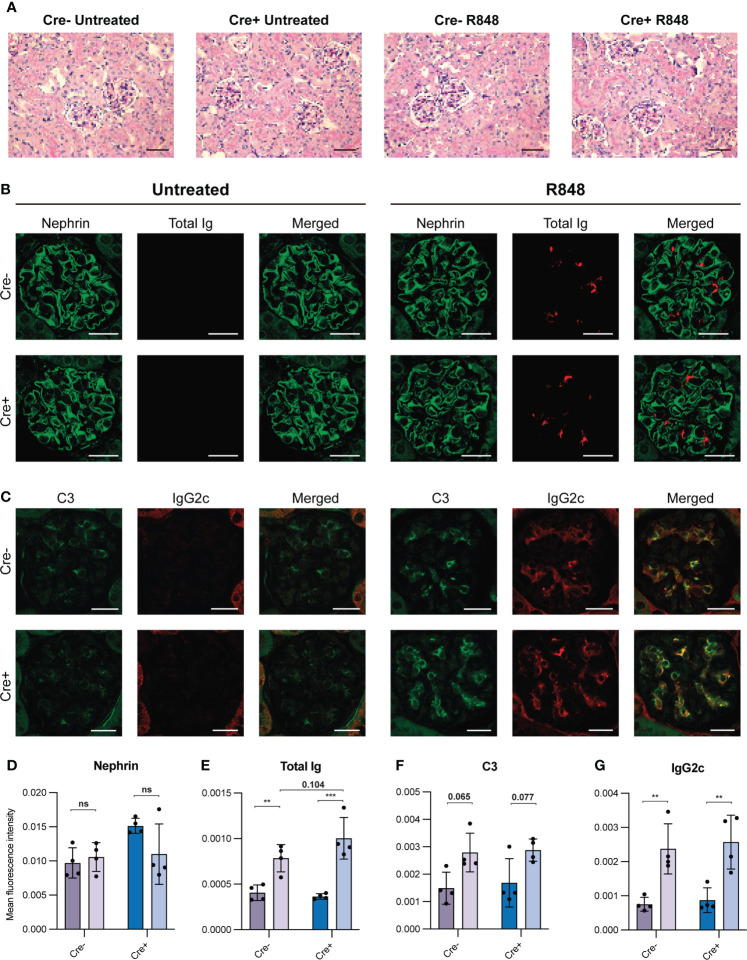
Kidney staining reveals immune complex deposition in R848-treated mice. **(A)** PAS stained kidney sections of Cre- untreated (n=4), Cre- R848-treated (n=4), Cre+ untreated (n=4), Cre+ R848-treated (n=4). Scale bar is 50 µm. **(B)** Immunofluorescence staining of kidney sections targeting nephrin (green) and total Ig (red). **(C)** Immunofluorescence staining of kidney sections targeting C3 (green) and IgG2c (red). **(D)** Quantification of immunofluorescence staining targeting nephrin, **(E)** total Ig, **(F)** C3, **(G)** and IgG2c. Scale bar is 20 µm. Two-way ANOVA with Holm-Sidak’s *post hoc* test was used to analyze the data. Bars show mean ± SD. ns = p>0.05, **p < 0.01, ***p < 0.001.

**Figure 4:**
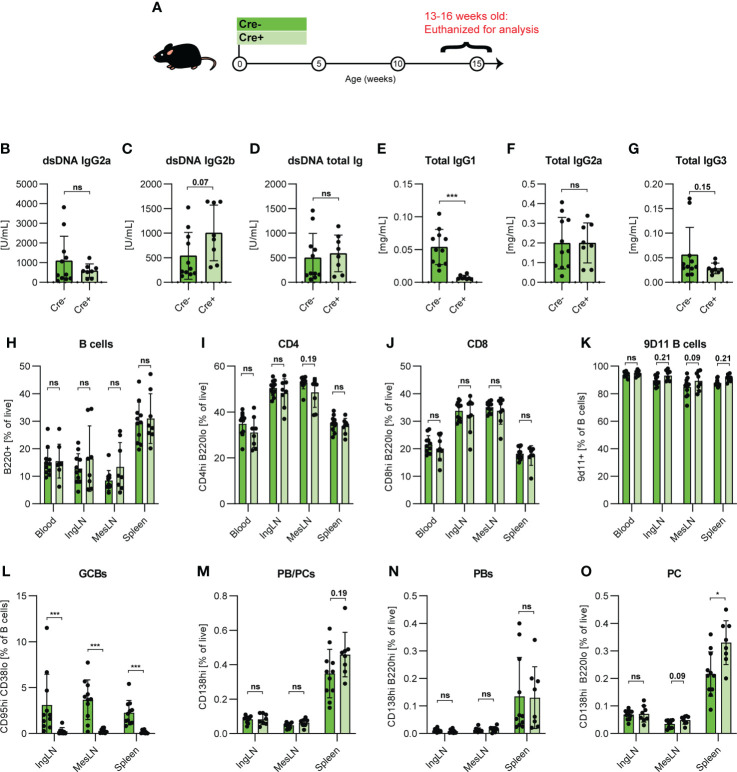
GC block causes increased PB/PCs levels in adolescent 564Igi mice. **(A)** Schematic overview of experimental setup: 564Igi-Bcl-6^flx/flx^ (Cre-, dark green, n=11) and 564Igi-Aicda-Cre Bcl-6^flx/flx^ (Cre+, light green, n=8) mice. **(B)** Anti-dsDNA IgG2a. **(C)** Anti-dsDNA IgG2b. **(D)** Anti-dsDNA total Ig. **(E)** Total IgG1. **(F)** Total IgG2a. **(G)** Total IgG3. Unpaired t-test or Mann-Whitney’s test was used was used to analyze TRIFMA data. **(H)** Flow cytometry analyses of B cell frequencies (B220^+^ CD4^-^ CD8^-^ of live, singlet lymphocytes) in blood, IngLN, MesLN, and spleen. **(I)** As H, but CD4 T cells (CD4^+^B220^-^). **(J)** As H, but CD8 T cells (CD8^+^B220^-^). **(K)** As H, but 9D11 B cell frequencies (9D11^+^). **(L)** As H, but GCB frequencies (CD95^hi^CD38^lo^). **(M)** As H, but PB and PC frequencies (CD138^hi^), **(N)** As H, but PB frequencies (CD138^hi^B220^hi^), **(O)** As H, but PC frequencies (CD138^hi^B220^lo^). Data are pooled from two independent experiments. Bar graphs show mean ± SD. Two-way ANOVA with Holm-Sidak’s *post hoc* test was used to analyze the data. ns = p≥0.05, *p < 0.05, ***p < 0.001.

**Figure 5:**
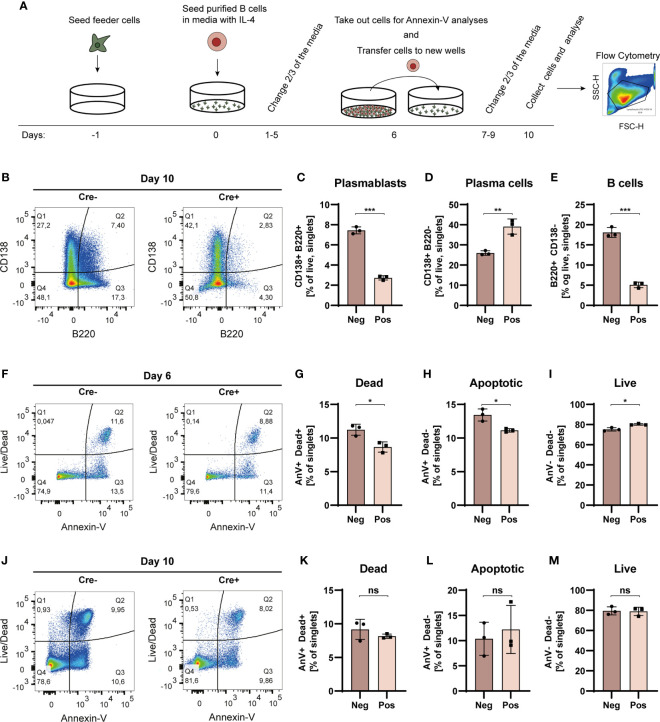
Bcl-6 deficient cells more readily differentiate into PCs in iGB cultures. **(A)** Schematic overview of the iGB culture system. **(B)** Representative terminal CD138 vs. B220 bivariate plot for iGB cultured B cells stimulated with IL-4. **(C)** Bar graph showing PB frequencies (B220^+^CD138^+^). **(D)** As C, but showing PC frequencies (B220^neg^CD138^+^). **(E)** As for C, but showing B cell frequencies (B220^+^CD138^neg^). Data are representative of three independent experiments with a cumulative 8 replicates in total. Bar graphs show mean ± SD. **(F)** Day 6 representative terminal Live/Dead vs. Annexin V bivariate plot for iGB cultured B cells. **(G)** Bar graph showing dead cell frequencies (AnV^+^Dead^+^). **(H)** As G, but showing apoptotic cell frequencies (AnV^+^Dead^neg^). **(I)** As G, but showing live cell frequencies (AnV^neg^Dead^neg^). **(J)** Day 10 representative terminal Live/Dead vs. Annexin V bivariate plot for iGB cultured B cells. **(K)** Bar graph showing dead cell frequencies (AnV^+^Dead^+^). **(L)** As K, but showing apoptotic cell frequencies (AnV^+^Dead^neg^). **(M)** As K, but showing live cell frequencies (AnV^neg^Dead^neg^). Data for apoptosis assays are from one experiment with 3 replicates. Bar graphs show mean ± SD. Unpaired t-tests were used to analyze all datasets. ns = p≥0.05, *p < 0.05, **p < 0.01, ***p < 0.001.

**Figure 6:**
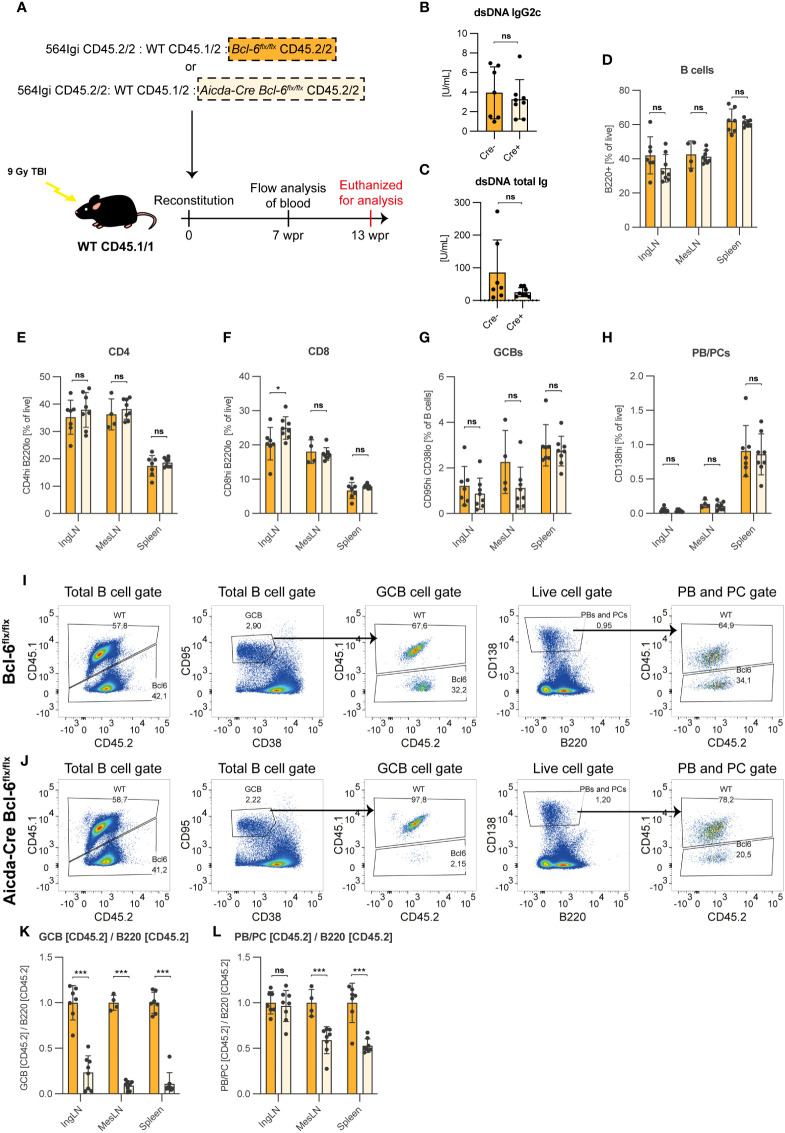
B cells harboring a GC block contribute to the PC lineage in a GC sufficient environment. **(A)** Schematic overview of the mixed bone marrow chimera setup. Lethally irradiated CD45.1/1 recipients were reconstituted with CD45.2/2 564Igi BM, WT CD45.1/2 BM and either Bcl-6^flx/flx^ (Cre-, orange, n=7) or Aicda-Cre Bcl-6^flx/flx^ (Cre+, light orange, n=8) (both CD45.2/2). **(B)** dsDNA IgG2c TRIFMA. **(C)** dsDNA total Ig TRIFMA. **(D)** Flow cytometric analysis of B cell frequencies (B220^+^ of live, singlets). **(E)** CD4 frequencies (CD4 of live, singlets). **(F)** CD8 frequencies (CD8 of live, singlets). **(G)** GCB frequencies (CD95^hi^ CD38^lo^ of B cells). **(H)** PB/PC frequencies (CD138^hi^ of live, singlets). **(I)** Representative bivariate plots with gates for Bcl-6^flx/flx^ chimeras. **(J)** Representative bivariate plots with gates for Aicda-Cre Bcl-6^flx/flx^ chimeras. **(K)** Ratio of CD45.2+ of GCB to CD45.2+ of total B cells. **(L)** Ratio of CD45.2+ of PBs/PCs to CD45.2+ of total B cells. The results are obtained from a single experiment with the number of mice given above. Bar graphs show mean ± SD. ns = p≥0.05, *p < 0.05, ***p < 0.001.

For the included mouse experiments, the experimental unit was a single animal. Cre- littermate groups were used as controls for Cre+ groups to determine impact of GC block, and untreated groups were used as controls for R848 treated groups to determine effect of R848 treatment. No *a priori* sample size determination was employed. The number of animals in each group is given in figure legends throughout the manuscript and supplement. Total number of animals for R848 cohort was n=29 (**Figure 1**), and a subset hereof were analyzed in **Figure 2** (n=25), **Figure 3** (n=16). Total number of animals for 564Igi adolescent cohort was n=19 (**Figure 4**). Total number of animals for 564Igi adult cohort was n=22 (**Figure S4**). Two independent animals per group were used for B cell purification and *in vitro* cultures presented in **Figure 5**. Total number of animals for mixed bone marrow chimeras was n=15 (**Figure 6**). No criteria for exclusion of animals were established *a priori*, and no animals were excluded from any of the cohorts. We did not perform randomization, rather group assignment was defined based on Cre+ or Cre- status, and selection for R848 treatment was based on minimal cage number (i.e., untreated and treated mice were in separate cages to avoid cross-contamination, and whereas re-caging was straightforward for females, males were divided based on minimal cage number required to balance untreated and treated groups), and with a view to sex balance (please refer to **Tables S1**, **S2**). Confounders were not controlled, and data were not blinded for analysis. Outcome measures were the read-outs presented in cohort-associated figures.

GraphPad Prism v. 8.4.3 was used for statistical analyses. Both tests for normality and Q-Q plots were used to determine whether data were normally distributed. Data that were not normally distributed were log-transformed and re-tested for normality. The following data sets were log-normally distributed: **Figures 1B, D, E, N, P, Q**, **3F**, **4H, L–O**, **6C, F–H**, **S4L–O**, **S7C**. For **Figures 4B, G**, **S4D, E, H**, Mann-Whitney test was used to analyze the data that were not normally distributed. All other datasets were normally distributed, except for the data in **Figure 1L**, **5F**, **S1B** (Spleen), **S1C** (IngLN), **S4K**, and **S7A**, which were neither normally, nor log-normally distributed. However, a non-parametric t-test for the isolated data between each group in panel 1L, **5F**, **S1B** (Spleen), **S1C** (IngLN) and **S4K** showed similar results (For **Figure 1L**: a significant increase in GC formation in Cre- between Unt and R848. For **Figure 5F**: a significant increase in CD8 frequencies in the IngLNs but not in other tissues. For **S1B** (Spleen): a significant increase between Cre- R848 and Cre+ R848. For **S1C** (IngLN): small, but significant increases with the results being more significant between Cre- Unt and Cre- R848. For **S4K**: slight, but significant increases across spleen, IngLN and blood, with a trend towards an increase in MesLN when tissues were analyzed individually), indicating that the observed differences, beyond being biologically robust and in agreement with the flow data, were also statistically robust. Parametric tests were used in all analyses, with specific tests indicated in the figure legends. All data is presented as bar graphs with mean ± SD, except for **S5A**, where we plotted the mean ± 95% CI, as a basis for statistically robust comparison of the means. All p-values were adjusted for multiple comparisons, and a p-value <0.05 was considered to be statistically significant. ns = p≥0.05, * = p<0.05, **= p<0.01, *** = p<0.001.

## Results

### Genetic GC block fails to curb autoimmune progression in a pharmacological SLE model

Enhanced Toll-like receptor-7 (TLR7) signaling is an established driver of SLE in mice (13) and humans (35). Chronic epicutaneous application of the synthetic, small-molecule TLR7 agonist, R848 (Resiquimod), induces a robust SLE-like autoimmune phenotype in multiple genetic background strains of *Mus musculus* (36). Leveraging this model, we set out to investigate the relative importance of the extrafollicular and GC pathways in the autoimmune response. To this end, we employed a transgenic Cre driver line displaying expression of Cre under the Aicda promotor (31). We combined this with a conditional Bcl-6 knock-out line (30), to achieve deletion of Bcl-6 specifically in GC B cells, without affecting Bcl-6 dependent T cell subsets essential to support both GC and extrafollicular responses (37). As controls, we employed Cre negative Bcl-6^flx/flx^ littermates. Previously, treatment of mice with R848 3 times per week for 8 weeks caused severe kidney damage, and 12 weeks of treatment led to a dramatic degree of mortality (36). To investigate the development of early immunological and histopathological hallmarks of disease in the absence of secondary effects caused by organ failure, we treated mice 3 times weekly for only 4 weeks (**Figure 1A**).

After 4 weeks of treatment with R848, Bcl-6^flx/flx^ (Cre-) controls and Aicda-Cre Bcl-6^flx/flx^ (Cre+) mice showed similar, significant increases in spleen weight (**Figure 1B**). Anti-dsDNA autoantibodies of IgG2c subtype were dramatically elevated in sera of R848 treated animals, and surprisingly were not reduced in treated Cre+ mice compared with treated Cre- mice (**Figure 1C**). A similar picture was seen for total IgG2c levels (**Figure 1D**). No statistically significant differences in total IgG1 and total IgG3 were seen upon treatment, but baseline IgG1 levels were lower in Cre+ mice (**Figures 1E, F**).

To validate the effect of R848 treatment and the integrity of the GC block in Cre+ mice, we carried out immunofluorescence staining of spleens (**Figures 1G–J**). Using the proliferation marker Ki67 and the naïve B cell marker IgD, it was evident that larger and more frequent GCs were observed upon R848 treatment in Cre- animals (**Figures 1G, H, K**), and this difference was statistically significant (**Figure 1L**). Cre+ animals did not display any baseline or R848-induced GCs (**Figures 1I, J**). However, in Cre+ treated mice we did observe many proliferating cells at the T-B border and in the red pulp (**Figure 1J**), likely abortive primary foci and extrafollicular foci, respectively.

Flow cytometric analyses of inguinal LNs (IngLNs), mesenteric LNs (MesLN), and the spleen were carried out (**Figures 1M–Q**, **Figure S1A**). In treated animals, we saw slight increases in monocyte and neutrophil frequencies in some tissues (**Figures 1M, N**), and a slight increase in B cell frequencies in skin-draining IngLNs (**Figure 1O**), maybe caused by the direct stimulatory effect from the R848 treatment of the ear skin. We observed robust GC B cell frequencies in Cre- R848 treated mice, compared to untreated littermates (**Figure 1P**). No GCBs were found in Cre+ animals, further validating the fidelity of the GC block (**Figure 1P**). Strikingly, despite this, we found a significant increase in PB/PC frequencies upon treatment in both groups, and there was a trend towards a higher PB/PC frequency in spleens of mice harboring a GC block compared to Cre- R848-treated littermate controls (**Figure 1Q**). This difference between the two groups for the spleen was attributable to PB rather than PC expansion in Cre+ animals, compared to Cre- littermates (**Figures S1B, C**). Taken together, this surprisingly indicated an unabated autoimmune phenotype in GC blocked compared to sufficient mice upon R848 treatment.

To further understand the local effects of R848 treatment, we performed immunofluorescence microscopy analyses of draining auricular lymph nodes (AurLNs) from treated mice and untreated controls. We observed gross enlargement of the lymph nodes of treated animals, with a robust induction of GCs in Cre- R848-treated mice (**Figure S2A**). In comparison, Cre+ R848-treated mice had many proliferating cells outside the follicles (**Figure S2A**). These dividing cells in the AurLNs overlapped to some extent with the PC marker CD138, pointing towards dividing extrafollicular PCs (**Figure S2B**).

### GC block does not mitigate the formation of superoligomeric DNA complexes

Using nanoparticle tracking analyses, we recently identified unique superoligomeric complexes (spMBL) formed between cell-free DNA and mannan-binding lectin (MBL), as a hallmark in blood samples from SLE patients and mice (33). These spMBL complexes correlated with disease activity in SLE patients, and correlated with formation of GCs and drove loss of immunological tolerance in a murine SLE model (564Igi). To further understand the importance of the increased anti-dsDNA IgG2c autoantibodies in Cre+ R848-treated mice, in the face of a complete absence of GCs, we implemented this nanoparticle tracking approach (**Figures 2A–C**). We analyzed superoligomeric complexes in the band from 90-130 nm (**Figures 2D, E**). In agreement with spMBL as an SLE marker, treated mice tended towards higher levels, as compared to untreated mice, but interestingly, we also observed a global trend towards higher levels in Cre+ compared to Cre- animals (**Figure 2F**). These observations were well in line with the previously noted increases in anti-dsDNA and total IgG2c antibodies upon R848 treatment, and in Cre+ compared to Cre- animals (**Figure 1C**).

In our prior study on autoimmune mice carrying an autoreactive B cell receptor knock-in (564Igi) on a wild-type background (33), a significant inverse correlation was found between the frequency of splenic GC B cells and the ratio between the spMBL and anti-dsDNA antibody concentrations measured in serum. This suggested that an excess of spMBL increased GCB formation while an excess of anti-dsDNA antibodies decreased GCB proliferation, or *vice versa*. This indicated a potential negative feedback loop, whereby complement activation driven by formation of spMBL-DNA complexes could be envisioned to exacerbate immune activation, whereas IgG produced by GC B cells would compete with MBL for DNA binding and complex formation or feedback-inhibit GC B cells, as previously suggested (38). In consideration of this, we next asked whether the same correlation could be established in the R848 model, and how this phenomenon was impacted in GC blocked mice.

In Cre- treated mice, GCB frequencies were positively correlated with the concentration of spMBL particles in serum (**Figure 2G**). There was no correlation between total IgG2c levels and spMBL particles (**Figure 2H**), but a significant positive correlation between anti-dsDNA IgG2c and spMBL particles (**Figure 2I**), demonstrating specificity. A significant positive correlation was also observed between the frequency of splenic GCBs and the ratio between the spMBL and total IgG2c levels in serum (**Figure 2J**). Conversely, a significant inverse correlation was found between the frequency of splenic GCBs and the ratio between the spMBL and anti-dsDNA IgG2c antibody concentrations measured in serum (**Figure 2K**). Taken together, this revealed an uncoupling of the concentration of spMBL particles and GC B cell levels in Cre+ mice, indicating that the GC block failed to curb the production of spMBL complexes characteristic of SLE.

### Immune complex deposition in kidneys of R848-treated mice is not prevented by GC block

To understand the pathological relevance of the elevated serum IgG2c, anti-dsDNA IgG2c and spMBL levels, we investigated pathological changes in the kidneys. Periodic acid-Schiff (PAS) staining revealed no significant histopathologic findings in any of the mice (**Figure 3A**), nor did we observe any differences in glomerular nephrin levels among the groups, suggesting normal glomerular podocytes (**Figure 3B**). This verified our short-term treatment strategy in terms of the goal to investigate early immune-driven events in the absence of any secondary pathology.

To evaluate if immune complex deposition occurred in the kidney glomeruli, and if there were any differences between GC-sufficient and deficient groups, we performed immunofluorescence staining of kidney sections targeting total Ig, C3, and IgG2c (**Figures 3B, C, E–G**). The total Ig levels in glomeruli were clearly increased upon R848-treatment, and were not reduced in treated Cre+ mice compared with treated Cre- littermates (**Figures 3B, E**). We also found a statistically significant increase in antibodies of the pathogenic subtype IgG2c (**Figures 3C, G**) and a trend towards an increase in C3 deposition upon R848 treatment (**Figures 3C, F**). However, no differences in C3 or IgG2c were seen between Cre+ and Cre- R848-treated groups (**Figures 3F, G**).

Taken together, R848-treated mice displayed immune complex deposition in glomeruli, based on an increased level of C3, total Ig and IgG2c (**Figures 3E–G**). We corroborated this by peroxidase-staining, as a corollary to the immunofluorescence microscopy, confirming the glomerular changes in total Ig, C3 and IgG2c upon R848-treatment (**Figure S3**). These findings correspond to Class I pathology (minimal mesangial lupus nephritis) displaying mesangial immune deposits without mesangial hypercellularity (39).

### The extrafollicular pathway is also sufficient to drive autoimmunity in autoreactive B cell receptor knock-in mice

To evaluate the robustness of our central observation that extrafollicular responses were sufficient to compensate autoimmune progression in the face of a complete GC block, we tested this in a second, independent SLE model. The 564Igi model is a B cell receptor knock-in model, derived from a B cell clone isolated from an autoreactive F1 hybrid cross of Swiss Inbred (SWR) and New Zealand Black (NZB) mice (13, 40). The clone was originally screened for reactivity with ssDNA, but has been found to react more generally with ribonucleic acids and several ribonuclear proteins (22), a polyreactivity likely owing to a high cationic pI in its complementarity determining region (41). 564Igi mice present with spontaneous GCs and robust levels of circulating anti-DNA antibodies, but do not develop more severe hallmarks of disease until later in life (>9-12 months of age). We crossed the 564Igi line with Aicda-Cre Bcl6^flx/flx^ mice, and compared Cre+ and Cre- Bcl6^flx/flx^ 564Igi H^+/+^K^+/+^ offspring either at adolescence (13-16 weeks) (**Figure 4A**) or in early adulthood (17-23 weeks of age) (**Figure S4A**). At the earlier time point, the two groups had comparable levels of anti-dsDNA of IgG2a isotype (**Figure 4B**), whereas GC blocked 564Igi mice presented with slightly elevated levels of anti-dsDNA IgG2b, although this difference did not reach statistical significance (**Figure 4C**). Total anti-dsDNA levels were comparable between groups (**Figure 4D**). Total IgG1 was elevated in GC-sufficient littermates (**Figure 4E**), whereas total IgG2a (**Figure 4F**) and IgG3 (**Figure 4G**) were on par. By flow cytometry (**Figure S5**), we verified that main lymphocyte subsets were comparable between groups across all tissues (**Figures 4H–J**). Furthermore, the frequency of idiotype positive cells, i.e., cells carrying the knock-in B cell receptor (identified by staining with the anti-idiotype antibody 9D11) was comparable between groups (**Figure 4K**). GCs were absent in Cre+ mice (**Figure 4L**), but despite this, overall PB/PC frequencies (**Figure 4M**) and PB frequencies (**Figure 4N**) across tissues were not different between the two groups although a trend towards an increased PB/PC frequency was observed in the spleen of mice harboring a GC block. PC frequencies were similar in IngLN and MesLN, and interestingly, significantly elevated in the spleen of GC block mice (**Figure 4O**). Thus, overall, the GC block did not prevent any of the autoimmune read-outs. This was also the case at the later time point, where no gross differences were observed (**Figure S4**).

Taken together, this demonstrated that the GC block failed to curb the main hallmarks of autoimmunity presented by the 564Igi model, further corroborating our findings from the pharmacological R848 model.

### An intrinsic GC block drives B cell differentiation into terminally differentiated PCs

To investigate whether B cell intrinsic GC block affects their capacity to differentiate into PBs and PCs, we made use of a modified *in vitro* setup for induced GC B cell (iGB) cultures (34, 42) (**Figure 5A**). Naïve B cells purified from Cre- and Cre+ Bcl-6^flx/flx^ mice were seeded onto fibroblast feeder cells expressing CD40L, IL-21 and BAFF, and stimulated with IL-4 to induce robust expansion of B cells with a GC-like phenotype, followed by differentiation into PBs and finally PCs (34, 42).

Flow cytometric analyses revealed significantly higher B cell (B220+, CD138neg) frequencies in cultures derived from Cre- mice, compared to those derived from Cre+ mice (**Figures 5B, E** and **Figure S6A, B**). This was mirrored by a similar relative increase in PBs (B220+, CD138+) in Cre- cultures (**Figures 5B, C**), but a relative decrease in PCs (B220neg, CD138+) (**Figures 5B, D**). The total number of cells in the live gate for Cre- cultures was approximately 4 times higher than that of Cre+ cultures (Cre-: 140,000 vs. Cre+: 33,000).

To understand this difference in cell numbers, we investigated whether the Cre+, and hence Bcl-6 deficient, B cells had an increased propensity to undergo apoptosis, because Bcl-6 has previously been reported to suppress P53 and inhibit apoptosis in GC B cells (43). Somewhat surprisingly, we found that upon IL-4 stimulation, there was) a slight and significant drop in both apoptotic and dead cell frequencies (**Figures 5F–H**), with a corresponding increased relative frequency of live cells in Cre+ cultures compared to Cre- cultures on day 6 (**Figures 5F, I**). However, at day 10 there were no significant differences in live, apoptotic, nor necrotic cell frequencies between Cre- and Cre+ cultures (**Figures 5J-M**). Thus, apoptosis could not account for the dramatic difference in resulting cell numbers. Taken together, this suggested that the higher overall cell numbers in Cre- cultures was not simply a reflection of increased apoptosis among Bcl-6 deficient cells in Cre+ cultures, but rather represented an improved intrinsic proliferative potential of the Bcl-6 sufficient cells.

To test this, we labeled Cre+ and Cre- cells with a division dye, then tracked their cell division capacity in the iGB culture setup. As an internal control for non-dividing cells, we included wells with Cre+ or Cre- cells, which received conditioned medium from the feeder cell line, containing BAFF and IL-21, but did not receive any CD40L or IL-4, critical to induce cell division. Indeed, both Cre- and Cre+ cells displayed a single homogenous population with undiluted division dye signal at day 3 of culture with conditioned medium only (**Figure S7A**). In contrast, iGB cultured cells displayed robust cell division at day 3, with a peak around 4 cell divisions, but no difference was observed between Cre+ and Cre- cells (**Figure S7A**). At day 6, the cell division peak had shifted to 9 divisions, and still no difference was observed between Cre+ and Cre- cells, although at this time point, the division dye was so diluted for the most divided cells, that it became difficult to distinguish from background. Therefore, it was not possible to assess cell division at the final time point, day 10; however, we did verify a comparable degree of apoptosis in the cultures (**Figure S7B**). Whereas cell numbers were similar at day 3, there was a slightly higher, but non-significant, increase in cell numbers for Cre- compared to Cre+ at day 6, and a robust and statistically significant increase by day 10 (**Figure S7C**). Notably, the frequency of dead cells, as assessed by viability dye staining, did not significantly differ between Cre+ and Cre- cells at any of the time points (**Figure S7D**). Overall, the increased expansion of Cre- cells and the similar degree of apoptosis and cell death in Cre- and Cre+ cultures supported our notion of an increased proliferative potential of Cre- cells. However, because we could not directly observe a difference in degree of cell division, we cannot exclude the possibility that Bcl-6 deficient cells could have a higher rate of turnover *via* lytic cell death, which would not be observable by the analyses employed.

In summary, our iGB experiments revealed a vigorous expansion of B cells and PCs in Cre- cultures, but less pronounced PC differentiation, whereas Cre+ cultures conversely displayed a lesser degree of proliferation but more pronounced PC differentiation. Thus, B cells with an intrinsic GC block may differentiate quicker to PCs, and thereby lose their capacity to divide, in line with the established function of Bcl-6 in repressing upregulation of Blimp-1 (44). However, we cannot exclude the possibility that an increased rate of lytic cell death among Bcl-6 deficient cells may also contribute to the observed differences in cell numbers.

### B cells harboring a GC block competitively contribute to the PC compartment in an autoreactive setting

Our observation that Bcl-6 deficient B cells rapidly lost their replicative potential *in vitro* (**Figure 5**) was somewhat at odds with our *in vivo* observations where, if anything, Cre+ mice displayed a global increase in PB/PCs (**Figures 1Q**, **4O** and **S1B**). However, in mice displaying a global GC block the PC differentiation process might be dysregulated, potentially owing to loss of GC-derived antibody feedback, as previously suggested for GC B cells (38). Alternatively, the disproportionate extrafollicular differentiation could be due to a constant autoinflammatory drive, either independent of or dependent upon the global prevention of B cell differentiation down the GC pathway. To discriminate between these possibilities, we took advantage of a mixed bone marrow (BM) chimera model allowing interrogation of the competitive potential of B cells with a defined genetic defect in an SLE-like setting (22, 32). In this model, 564Igi cells initiate an autoreactive process that subsequently recruits proto-autoreactive B cells from the non-564Igi B cell population. The B cells derived from the 564Igi compartment eventually are outcompeted and constitute only a minor fraction of the total B cell repertoire. Uniquely to this model, the spontaneous autoreactive GCs established by the 564Igi compartment become populated and chronically self-sustained by the non-564Igi (WT) B cells and gain independence from the initial 564Igi trigger. From around six weeks after reconstitution, GCs are almost exclusively (~95%) composed of WT-derived cells (11, 22). Reconstitution with a third of each of 564Igi BM, BM from a wild-type donor, and BM from a donor harboring a specified genetic defect, results in chimeras with two equal-sized compartments of B cells sufficient or deficient in the gene of interest. With the use of appropriate congenic markers, their competitive recruitment and participation in the autoreactive GC reaction and relative contribution to the PC compartment can subsequently be evaluated to elucidate the functional relevance of their intrinsic molecular differences. Accordingly, we set up mixed chimeras by irradiating WT CD45.1/1 recipients (carrying the congenic marker CD45.1 on both alleles) and reconstituting with 1/3 of each of 564Igi knock-in BM (CD45.2/2), WT CD45.1/2 BM, and either Aicda-Cre+ or Aicda-Cre- Bcl-6^flx/flx^ BM (CD45.2/2) (**Figures 6A**, **S8**).

There were no gross differences in the basic parameters when comparing Cre- control chimeras with Cre+ chimeras, in which approximately 50% of the B cells harbored a GC block (**Figures 6B–H**). This confirmed that the two groups of chimeras were comparable and had robust GCB and PC compartments. In the total B cell compartment, CD45.2 single-positive cells (i.e., CD45.2/2) were present at levels comparable to that of CD45.1 positive cells (i.e., CD45.1/2 or CD45.1/1) in both Cre- (**Figure 6I**) and Cre+ (**Figure 6J**) chimeras. However, within the GCB gate, CD45.2 single-positive cells were robustly represented in Cre- chimeras, but virtually absent in Cre+ chimeras (**Figures 6I, J**). When quantifying this effect across chimeras and expressing as the ratio of CD45.2 of GCB relative to CD45.2 of total B cells, it was clear that Bcl-6 deficient cells were incapable of contributing to the GCB compartment (**Figure 6K**). However, when similarly comparing PB/PC ratio over B cells, the cells harboring a GC block remained able to contribute to the final PB/PC pool, albeit underrepresented relative to the competitor cells (**Figure 6L**).

These findings demonstrated that in a GC-sufficient environment, B cells experiencing a block in their ability to partake in the GC reaction readily contributed to the PB and PC compartments.

## Discussion

There has been extensive interest in targeting GCs in autoimmune disease due to their unique roles in potent antibody responses, memory generation, and long-lived PC formation. The strategy has proven useful in autoimmune models, but due to off-target effects, did not initially progress through clinical trials (45, 46). Considerable efforts have, however, been aimed at circumventing the off-target effects to bring this strategy to market (45, 47). This notwithstanding, a recent study reported that extrafollicular B cell differentiation into short-lived antibody-forming cells is a key mechanism of anti-DNA autoreactivity (48), and it has been suggested that more attention should be paid to the non-GC responses, as these may play a critical role in humoral immunity in both mice and men (49).

Here, we took an unbiased approach and asked to what extent a genetic GC block would ameliorate autoreactive manifestations in an SLE-like disease model. To our surprise, it did not lessen any of the investigated autoimmune manifestations (**Figures 1**–**3**). This indicated that rerouting of the pathogenic response *via* the extrafollicular pathway could compensate for a complete GC block. We corroborated this central finding in a second, independent model (**Figure 4**), confirming that the observed sufficiency of the extrafollicular response was not model-specific. Of note, both these models are highly dependent on TLR7 (13, 36), and target nucleic acid antigens, which may explain the different outcome compared to the collagen-induced arthritis model (29). However, these qualities of the employed models are reflective of human SLE (19, 35), giving credence to the translational potential of our observations.

To understand the B cell intrinsic effect of a block in GC differentiation, we leveraged an induced GC B cell (iGB) culture system. It has previously been noted that Bcl-6 expression can inhibit apoptosis in numerous cell types including (GC) B cells (43, 50, 51). Yet, contrary to expectations, GC blocked B cells did not display a significantly increased propensity to undergo apoptosis, rather, they much more readily underwent terminal differentiation to PCs, and had a dramatically reduced capacity to expand compared to their wild type counterparts (**Figure 5**, **Figure S7**). Nonetheless, we did not observe an increased ability of Cre- cells to divide at early time points (day 3 and 6) compared to their Cre+ counterparts, suggesting that this effect plays out later, that Bcl-6 deficient cells have an increased propensity to undergo lytic cell death, which we cannot detect by traditional viability assays, or a combination of the two (**Figure S7**). This notwithstanding, their unequal differentiation potential agreed well with the established cross-regulation between Bcl-6 and Blimp-1, the master regulator of the PC fate, also known as Prdm1 (44). Although the increased propensity for terminal PC differentiation was, in principle, well in line with our *in vivo* observations, the lack of proliferative capacity was at the same time at odds with the dramatic PC output in the mice harboring a GC block in B cells, suggesting that this loss of proliferative capacity was compensated for *in vivo* (**Figures 1Q**, **4O**, **S1B**). This suggested that the PC differentiation process in mice displaying a global GC block in B cells might be dysregulated, potentially as a consequence of absence of GC-derived antibody feedback, as previously suggested for GC B cells (38). To address this possibility, we asked whether B cells with a GC block would be precluded from contributing to the PC pool in a GC sufficient environment. Our findings demonstrated that this was not the case, although the relative contribution of GC blocked B cells to the PC pool was smaller than that of GC sufficient B cells (**Figure 6**). However, given their inability to expand in GCs, the magnitude of the contribution of GC blocked B cells to the PC compartment in direct competition with GC sufficient B cells was remarkable. In the infectious setting, an early wave of extrafollicular PCs is crucial for the initial antibody response. However, most PCs produced by the extrafollicular response undergo apoptosis within a matter of days, and the global response becomes dominated by GC-derived responses. In the chronic autoreactive setting, however, the continuous fueling of the autoimmune process may continually renew the extrafollicularly derived PB/PC population. This was even the case in the 564Igi model at 17-23 weeks of age, demonstrating that extrafollicular responses are not outpaced by GCs in a chronic response lasting 5-6 months (**Figures 4**, **S4**). However, at this point it still remains unclear whether the GC responses observed in our models contribute a qualitatively different response to the autoimmune progression, e.g., through production of memory B cells and long-lived PCs that may perpetuate and dominate the chronic response over longer periods of time. By extension, the GC pathway may differentially allow epitope spreading and inclusion of alternative antigens over time, as seen in human SLE patients (23). At least, it seems plausible that the longer the autoimmune process has persisted, the more the long-lived GC responses and their derived memory output come to dominate the process. However, conversely, the short-lived extrafollicular responses may govern the early stages of the response and, as previously suggested, the very early break-of-tolerance driven by nucleic acid-containing antigens (8, 48).

Due to its more potent nature, the GC reaction is believed to be subject to a much higher level of control, through a continued requirement for linked recognition in successive rounds of diversity generation. Furthermore, a specialized subset of Tregs, T_FR_s, exert a dominant negative level of control on the GC reaction (26). Hence it may be that the extrafollicular pathway in essence represents an evolutionary ‘backdoor to autoimmunity’, unguarded due to its relative insignificance in terms of high-quality, affinity-matured, and memory-inducing antibody responses. In this context, it is fortunate that current CD40L targeting strategies block both the GC and extrafollicular response. However, we suggest that future efforts should be aimed at further elucidating the relative contributions of the extrafollicular and GC pathways. We may speculate that specific targeting of the extrafollicular pathway would be a superior strategy, as it would preferentially block the low quality and poorly controlled responses driving autoimmune progression, while leaving intact the more stringently controlled and high-quality responses that provide protection against infectious agents. However, the extrafollicular pathway does also play a role in providing rapid protection against newly encountered pathogens (52), and consequently blocking this response could cause a vulnerability to new pathogens, necessitating careful consideration of such aspects in future work. Unfortunately, there is much more limited knowledge regarding the biology of the extrafollicular responses, and no transgenic or pharmacologic strategy allowing specific blockade of this pathway exists, making it difficult to evaluate in animal models.

In summary, our findings here demonstrate that a complete block of the GC pathway is insufficient to curb autoreactive PC differentiation and autoimmune progression. The GC commitment is controlled by the expression level of the master transcriptional repressor, Bcl-6 (53), which regulates the GC fates across GC B cells, T_FH_ cells and T_FR_ cells. Interestingly, in the context of the COVID-19 pandemic, it has been observed that Bcl-6^+^ GC B cells and Bcl-6^+^ T_FH_ cells are markedly diminished in SARS-CoV-2 infection (54). It has also been found that critically ill SARS-CoV-2 patients display hallmarks of extrafollicular B cell activation and shared B cell repertoire features previously described in autoimmune settings (55, 56). This further highlights the potential link between aberrant extrafollicular responses and autoimmune manifestations.

## Data availability statement

The raw data supporting the conclusions of this article have been deposited to Dryad, and can be accessed via the following link: https://doi.org/10.5061/dryad.zs7h44jc2.

## Ethics statement

The animal study was reviewed and approved by The Danish Animal Experiments Inspectorate. All animal experiments were conducted in accordance with European Community guidelines and were approved by the Danish Animal Experiments Inspectorate (protocol numbers 2017-15-0201-01348 and 2017-15-0201-01319).

## Author contributions

LV and SD conceived the study and its design. LV, AH, TW, SH, KJ-M, KK, MP, and LJ conducted experiments and collected data. LV, KJ-M, AP, TV-J, KW and SD analyzed and interpreted data. LV and KJ-M prepared the figures. LV and SD wrote the first draft of the manuscript. All authors contributed to the article and approved the submitted version.
