# Supplementary material for: The extrafollicular response is sufficient to drive initiation of autoimmunity and early disease hallmarks of lupus
Source: Front Immunol. 2022 Dec 14;13:1021370. doi: 10.3389/fimmu.2022.1021370 (PMC9795406; doi:10.3389/fimmu.2022.1021370)
Supplement: Supplementary file 1 [file DataSheet_1.docx]

**
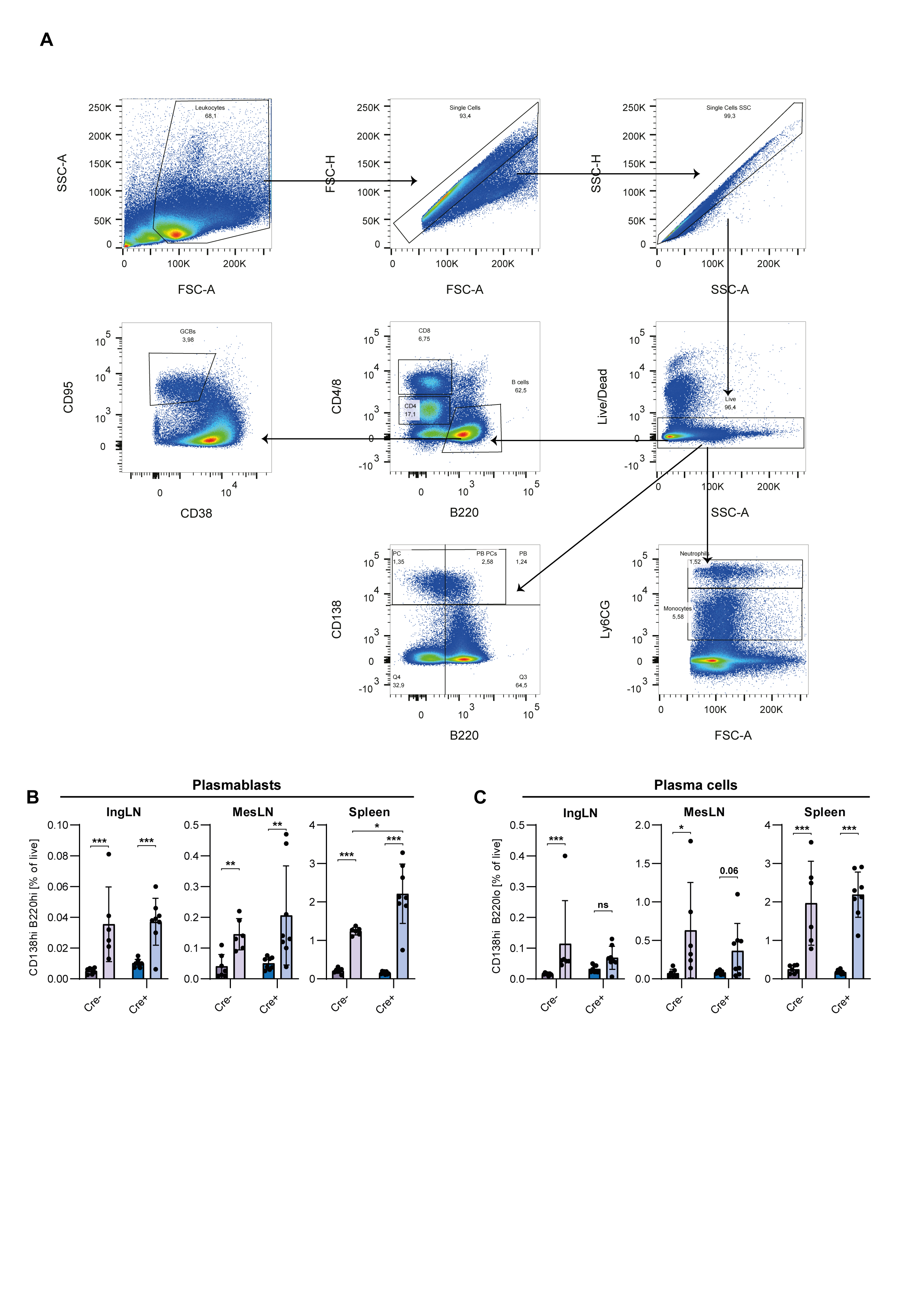
Supplementary Figure 1.** Gating strategy for R848 cohorts, PB and PC frequencies. (**A**) Leukocytes were gated based on size (FSC-A) and granularity (SSC-A). Doublets were excluded with two singlet gates, first FSC-H vs. FSC-A, and then SSC-H vs. SSC-A. After that, dead cells were excluded. From the live gate, monocytes and neutrophils were gated using the Ly6C/G marker. Moreover, B cells were selected, from which GC B cells were selected based on CD95 expression and the absence of CD38 expression. PB and PCs were selected from the live gate based on CD138. (**B**) PB (CD138^hi^ B220^hi^ of live, singlet leukocytes) frequencies across secondary lymphoid tissues. (**C**) PC (CD138^hi^ B220^lo^ of live, singlet leukocytes) frequencies across secondary lymphoid tissues. Data in B and C are pooled from two independent experiments. Bar graphs show mean $\pm$ SD. Two-way ANOVA with Holm-Sidak’s post hoc test was used to analyze the data. ns = p≥0.05, * = p<0.05, ** = p<0.01, *** = p<0.001.
