# Supplementary material for: The extrafollicular response is sufficient to drive initiation of autoimmunity and early disease hallmarks of lupus
Source: Front Immunol. 2022 Dec 14;13:1021370. doi: 10.3389/fimmu.2022.1021370 (PMC9795406; doi:10.3389/fimmu.2022.1021370)
Supplement: Supplementary file 2 [file DataSheet_2.docx]

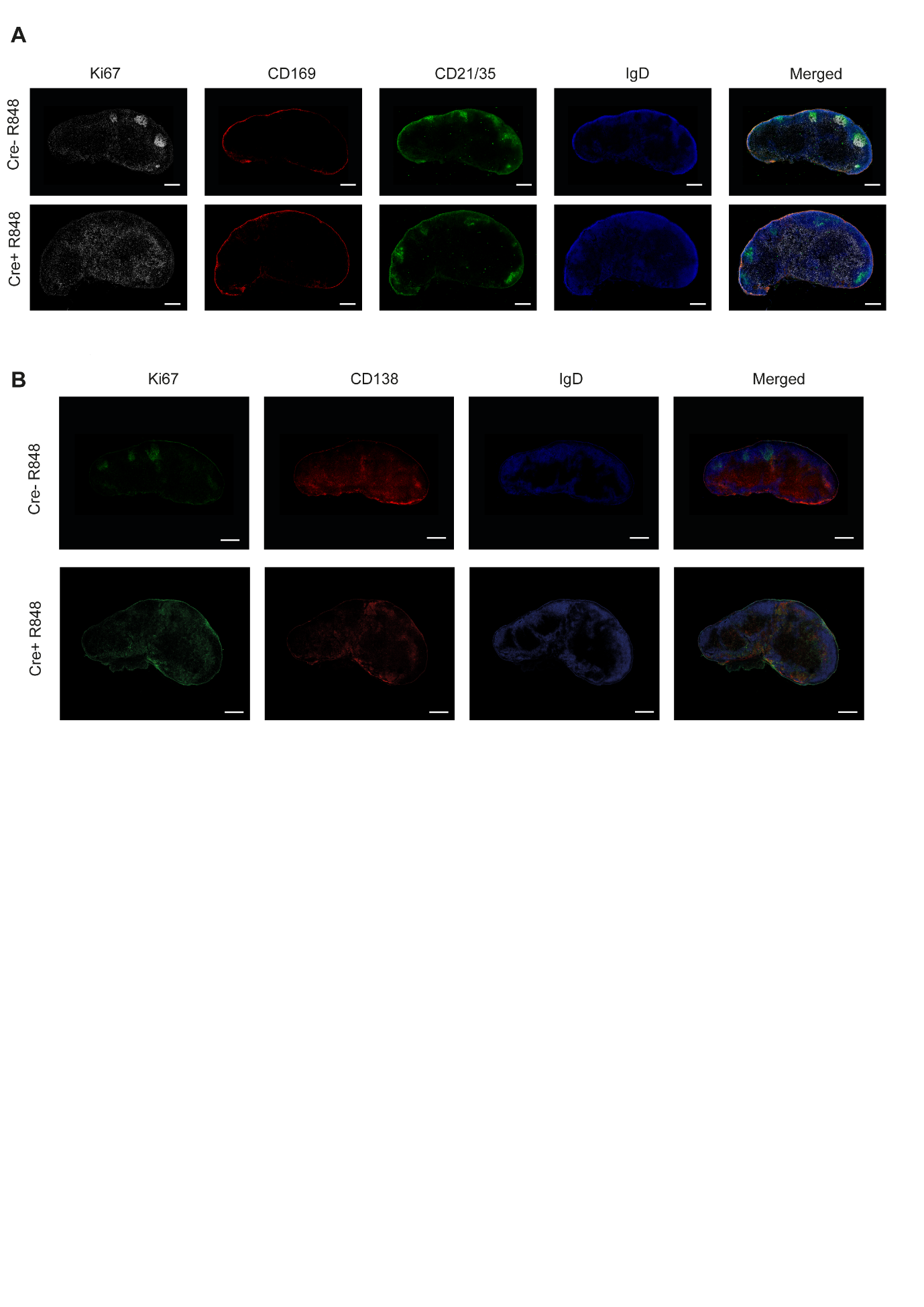


**Supplementary Figure 2.** Immunofluorescence stainings show dividing cells in the paracortex of the AurLN which overlaps with CD138 staining. (**A**) The following targets were stained for: Ki67 (white), CD169 (red), CD21/35 (green), IgD (blue). (**B**) The following targets were stained for: Ki67 (green), CD138 (red), IgD (blue). Scale bar is 350 μm. Images were generated by tile scanning with a 10% overlap using a mechanical stage, followed by stitching. Channel intensities have been adjusted for visual clarity.
