# Supplementary material for: The extrafollicular response is sufficient to drive initiation of autoimmunity and early disease hallmarks of lupus
Source: Front Immunol. 2022 Dec 14;13:1021370. doi: 10.3389/fimmu.2022.1021370 (PMC9795406; doi:10.3389/fimmu.2022.1021370)
Supplement: Supplementary file 3 [file DataSheet_3.docx]

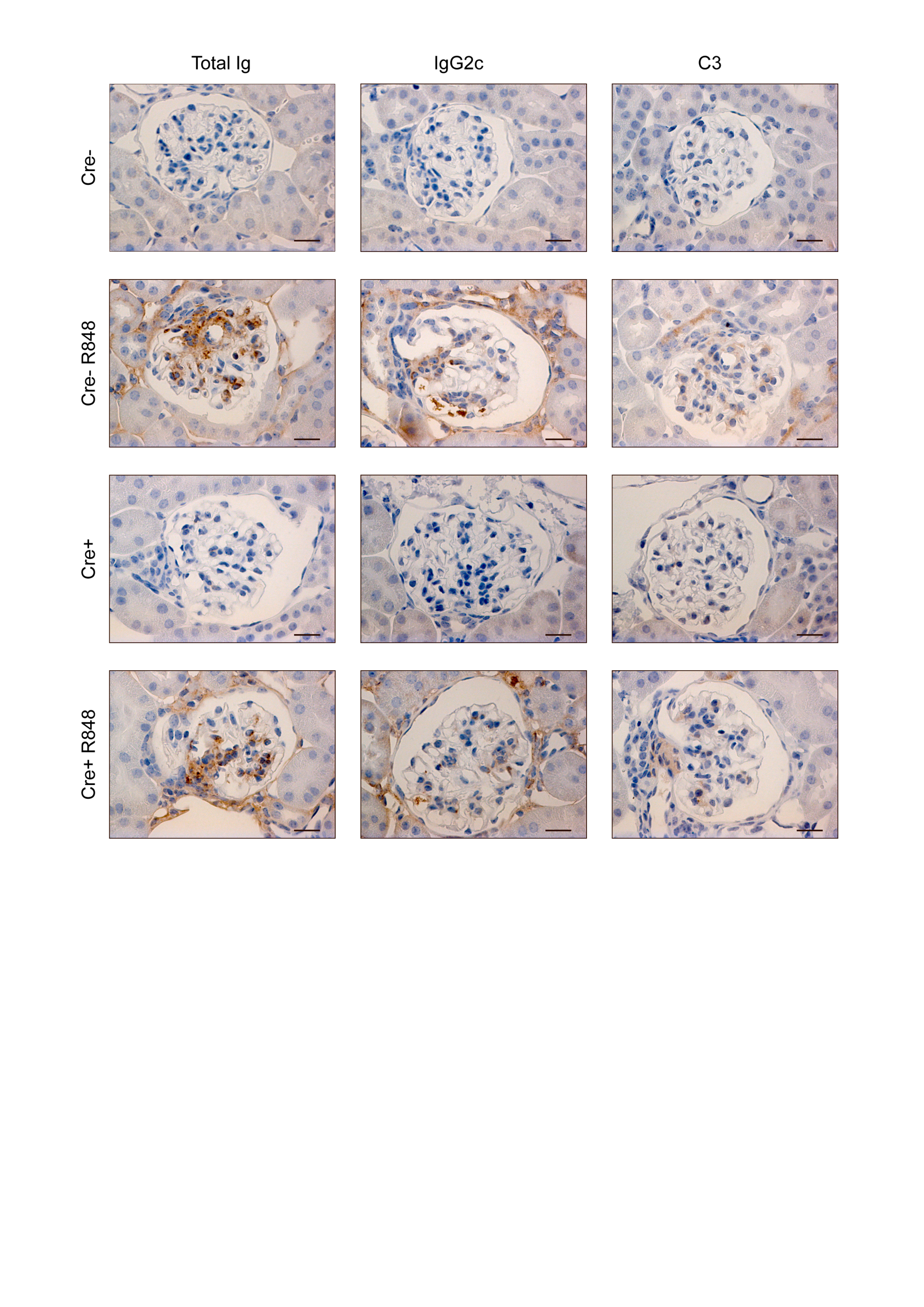


**Supplementary Figure 3.** Increased immune deposits in glomeruli of R848-treated mice. Representative images of immunohistochemical peroxidase staining targeting total Ig, IgG2c and C3 on sections from Cre- untreated (n=4), Cre- R848-treated (n=4), Cre+ untreated (n=4), Cre+ R848-treated (n=4) mice. Scale bars = 20 μm.
