# Supplementary material for: The extrafollicular response is sufficient to drive initiation of autoimmunity and early disease hallmarks of lupus
Source: Front Immunol. 2022 Dec 14;13:1021370. doi: 10.3389/fimmu.2022.1021370 (PMC9795406; doi:10.3389/fimmu.2022.1021370)
Supplement: Supplementary file 4 [file DataSheet_4.docx]

**
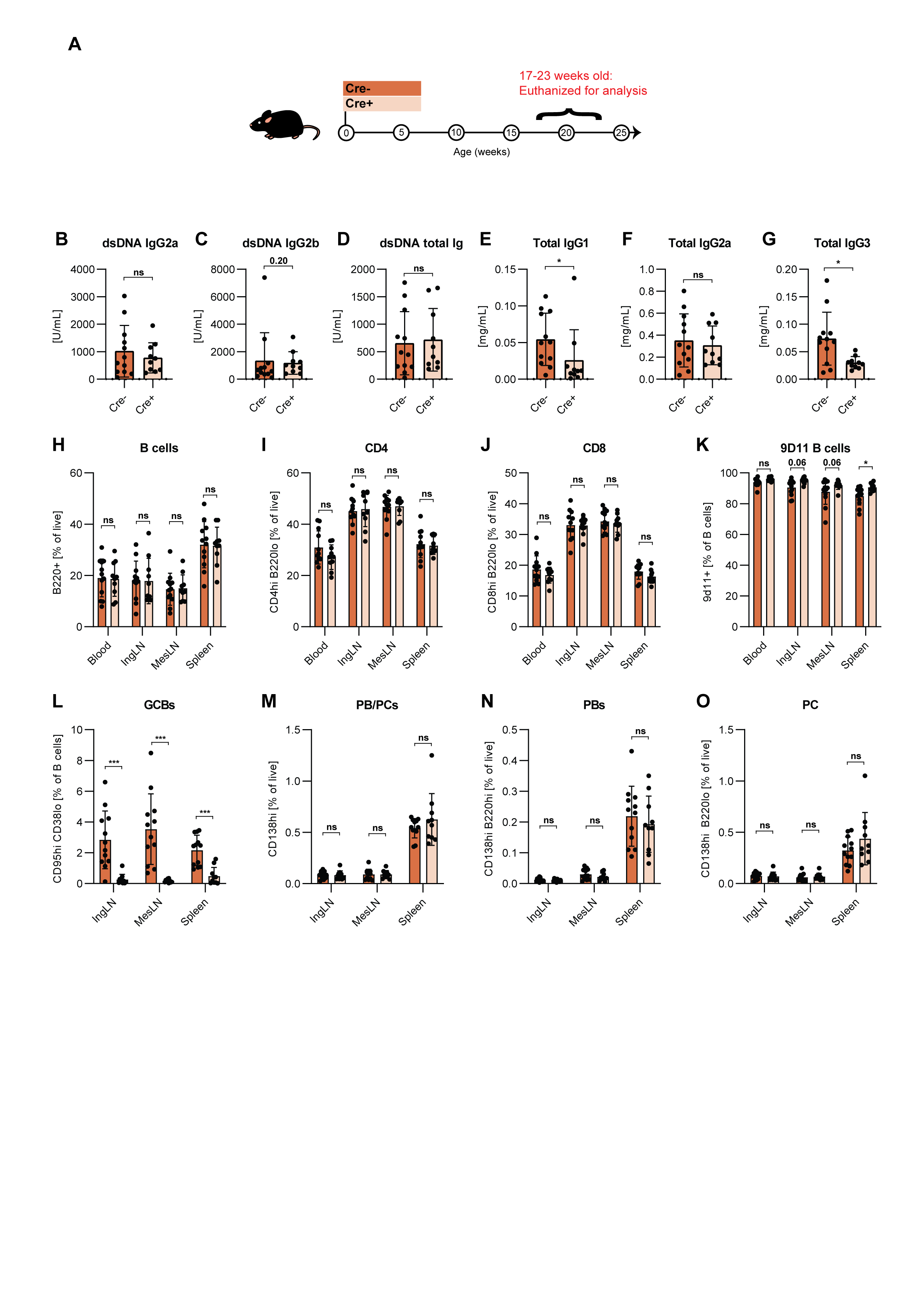
Supplementary Figure 4.** GC block does not ameliorate autoimmune phenotype in adult 564Igi mice. (**A**) Schematic overview of experimental setup: 564Igi-Bcl-6^flx/flx^ (Cre-, dark red, n=12) and 564Igi-Aicda-Cre Bcl-6^flx/flx^ (Cre+, light red, n=10). (**B**) Anti-dsDNA IgG2a. (**C**) Anti-dsDNA IgG2b. (**D**) Anti-dsDNA total Ig. (**E**) Total IgG1. (**F**) Total IgG2a. (**G**) Total IgG3. Unpaired t-test or Mann-Whitney’s test was used was used to analyze TRIFMA data. (**H**) Flow cytometry analyses of B cell frequencies (B220^+^ CD4^-^ CD8^-^ of live, singlet lymphocytes) in blood, IngLN, MesLN, and spleen. (**I**) As H, but CD4 T cells (CD4^+^ B220^-^ of live, singlet lymphocytes). (**J**) As H, but CD8 T cells (CD8^+^ B220^-^ of live, singlet lymphocytes). (**K**) As H, but 9D11 B cell frequencies (9D11^+^ of B cells). (**L**) As H, but GCB frequencies (CD95^hi^ CD38^lo^ of B cells). (**M**) As H, but PB and PC frequencies (CD138^hi^ of live, singlet lymphocytes), (**N**) As H, but PC frequencies (CD138^hi^ B220^lo^ of live, singlet lymphocytes), (**O**) As H, but PB frequencies (CD138^hi^ B220^hi^ of live, singlet lymphocytes). Data are pooled from two independent experiments. Bar graphs show mean $\pm$ SD. Two-way ANOVA with Holm-Sidak’s post hoc test was used to analyze the data. ns = p≥0.05, * = p<0.05, ** = p<0.01, *** = p<0.001.
