# Supplementary material for: The extrafollicular response is sufficient to drive initiation of autoimmunity and early disease hallmarks of lupus
Source: Front Immunol. 2022 Dec 14;13:1021370. doi: 10.3389/fimmu.2022.1021370 (PMC9795406; doi:10.3389/fimmu.2022.1021370)
Supplement: Supplementary file 6 [file DataSheet_6.docx]

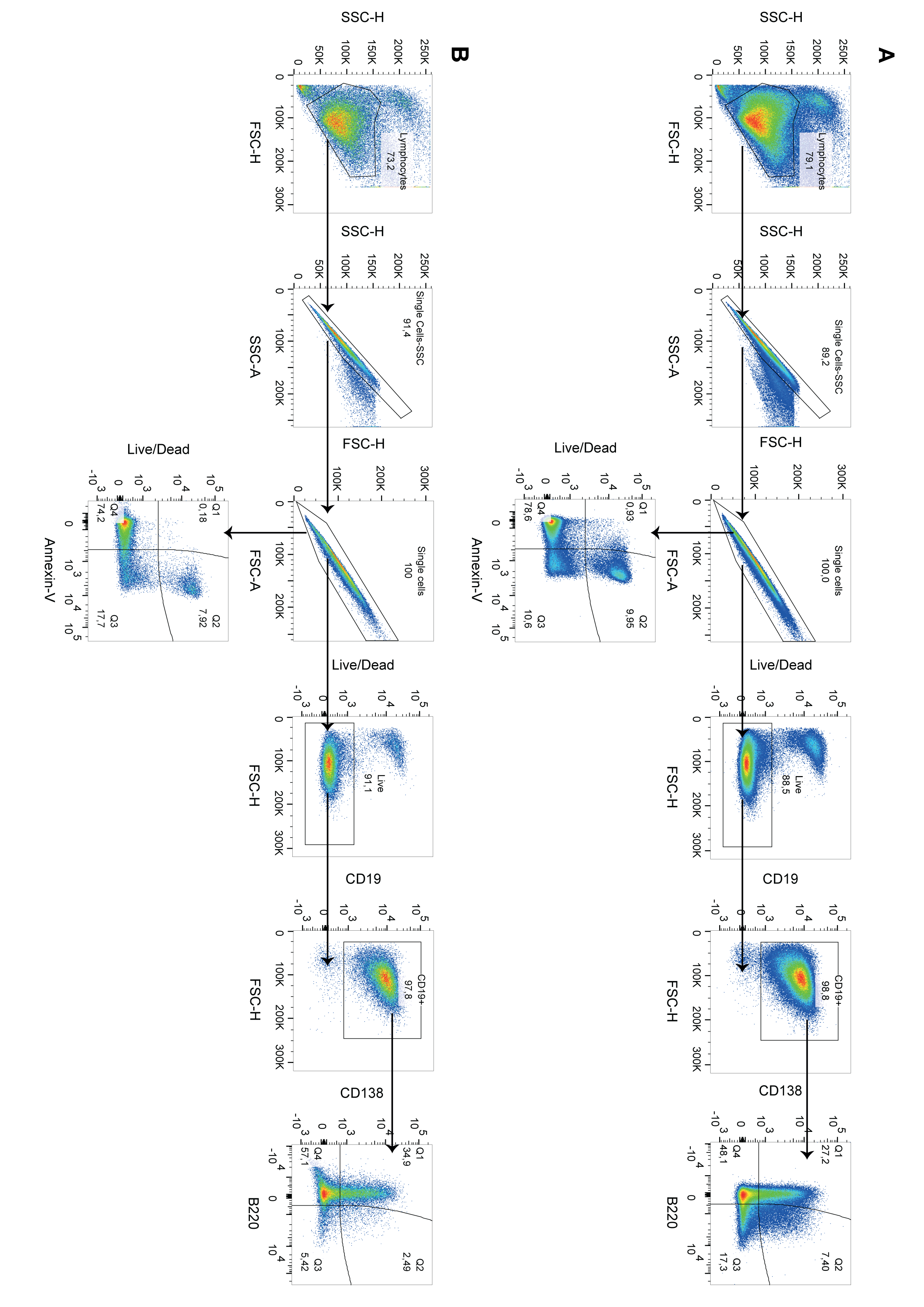


**Supplementary Figure 6.** Gating strategy for Nojima cultures for (**A**) Cre-, (**B**) Cre+. Lymphocytes were gated based on size (FSC-H) and granularity (SSC-H). Doublets were excluded with two consecutive singlet gates: 1) SSC-H vs. SSC-A, and 2) FSC-H vs. FSC-A. Apoptotic cells were gated based on the singlet gate using Annexin V and live/dead marker. Dead cells were excluded and CD19+ cells were afterwards selected. From this, PCs and PBs were gated based on CD138 and B220 levels.
