# Supplementary material for: The extrafollicular response is sufficient to drive initiation of autoimmunity and early disease hallmarks of lupus
Source: Front Immunol. 2022 Dec 14;13:1021370. doi: 10.3389/fimmu.2022.1021370 (PMC9795406; doi:10.3389/fimmu.2022.1021370)
Supplement: Supplementary file 7 [file DataSheet_7.docx]

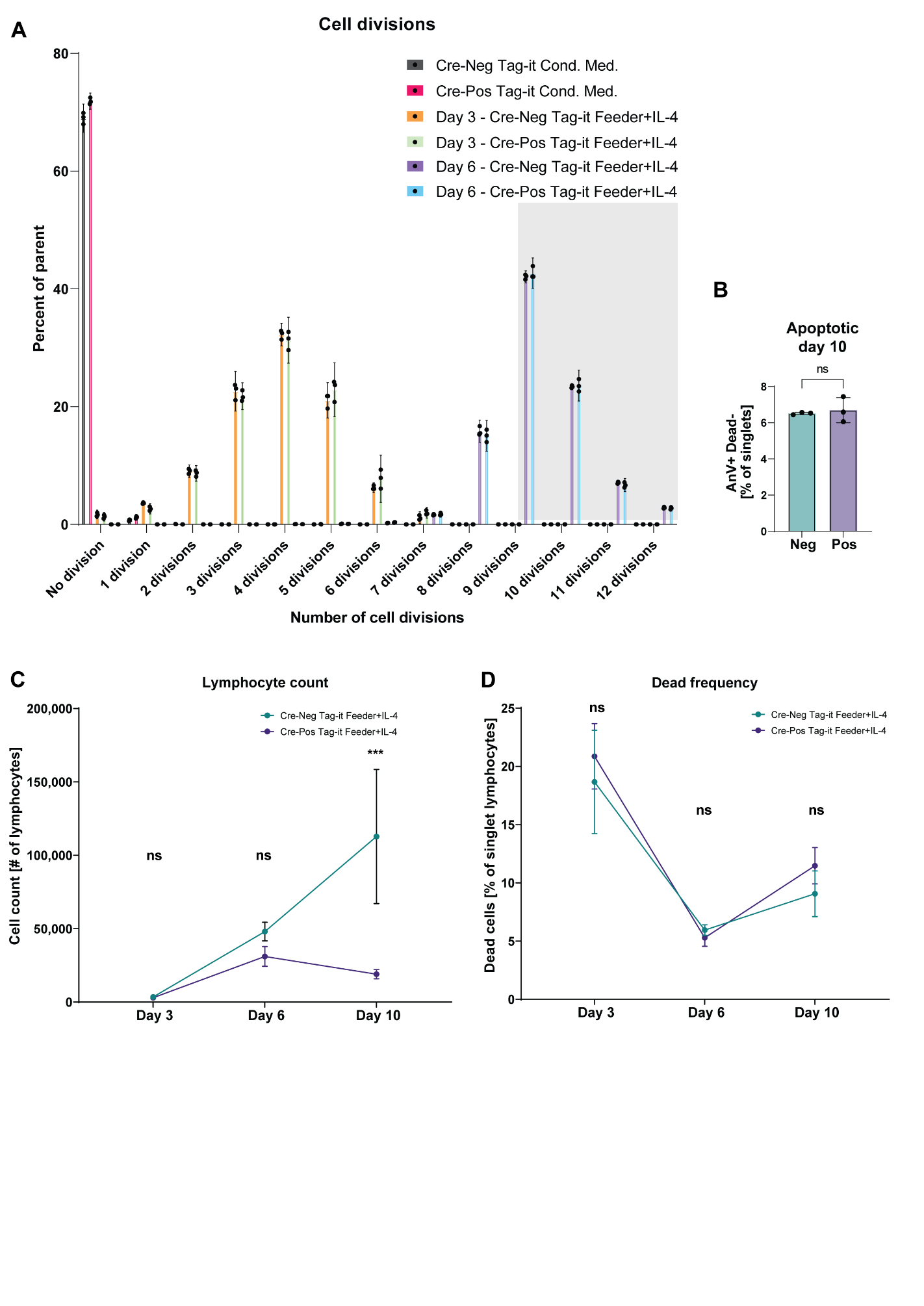


**Supplementary Figure 7.** GC blocked B cells do not divide more than GC sufficient cells in iGB cultures at day 3 and 6, but have proliferated to a greater extent by day 10. (**A**) Cell division assay for B cells in conditioned media at day 3 (Cre neg: black bars, Cre pos: pink bars), B cells with feeder cells + IL-4 at day 3 (Cre neg: orange bars, Cre pos: green bars), or B cells with feeder cells + IL-4 at day 6 (Cre neg: purple bars, Cre pos: blue bars). Division 9 – 12 are greyed out, because the MFIs for cells displaying this degree of division began approaching the MFI of the background, making it difficult to quantify precisely. Bar graphs show mean±95% CI. (**B**) Flow cytometry analysis of apoptosis at day 10 in cell division tracked wells. Bars represent mean±SD. (**C**) As B, but for lymphocyte counts at day 3, 6, and 10. (**D**) As B, but for frequency of dead cells at day 3, 6, and 10.
