# Supplementary material for: The extrafollicular response is sufficient to drive initiation of autoimmunity and early disease hallmarks of lupus
Source: Front Immunol. 2022 Dec 14;13:1021370. doi: 10.3389/fimmu.2022.1021370 (PMC9795406; doi:10.3389/fimmu.2022.1021370)
Supplement: Supplementary file 8 [file DataSheet_8.docx]

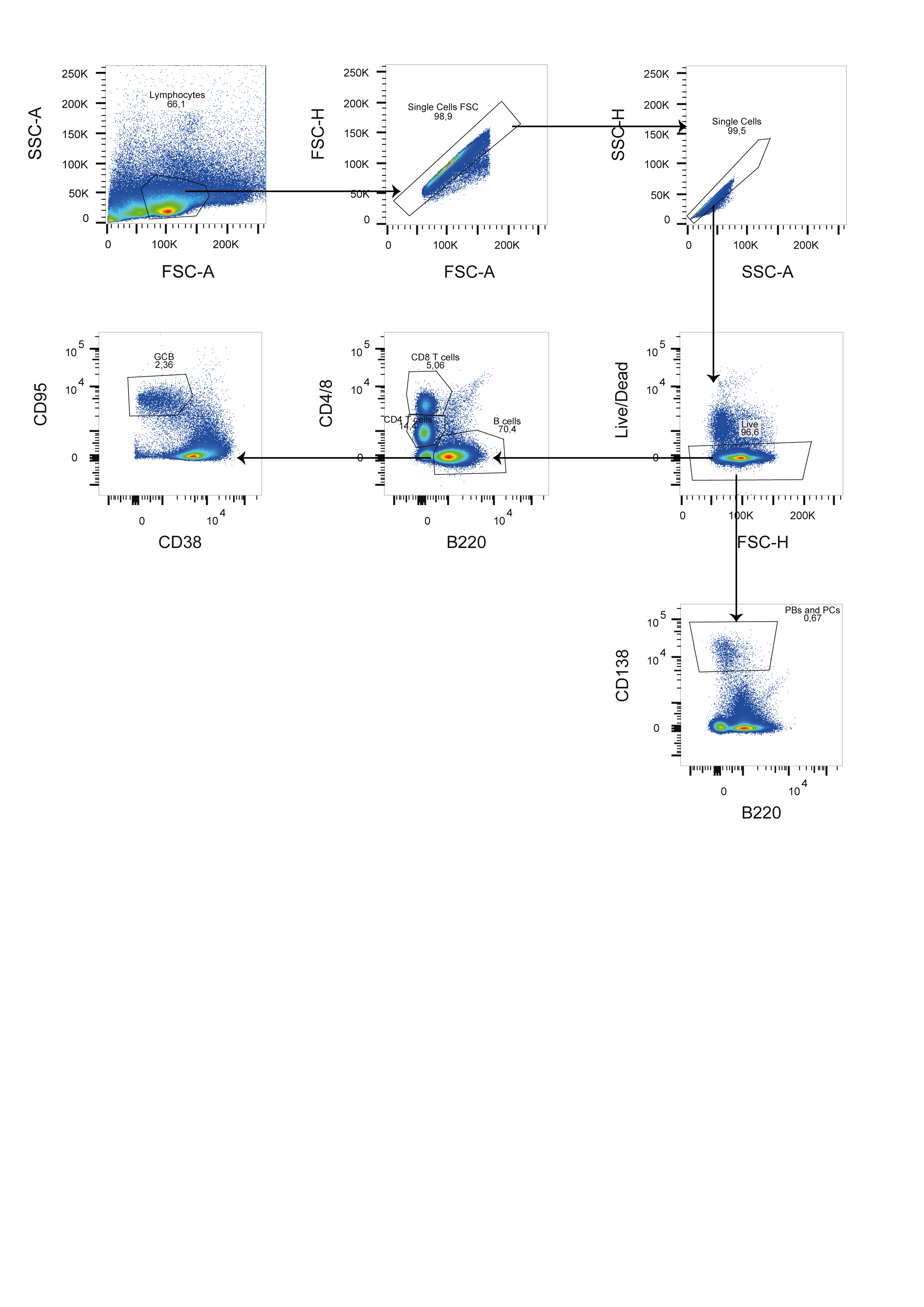


**Supplementary Figure 8.** Gating strategy for the mixed bone-marrow chimera model cohort: Lymphocytes were gated based on size (FSC-A) and granularity (SSC-A). Doublets were excluded with two singlet gates, first FSC-H vs FSC-A, and then SSC-H vs. SSC-A. Dead cells were excluded. B cells were selected from the live gate based on B220+ expression, from which GCBs were selected based on CD95 expression and the absence of CD38 expression. PBs and PCs were selected from the live gate based on CD138 expression.
