# Supplementary material for: The extrafollicular response is sufficient to drive initiation of autoimmunity and early disease hallmarks of lupus
Source: Front Immunol. 2022 Dec 14;13:1021370. doi: 10.3389/fimmu.2022.1021370 (PMC9795406; doi:10.3389/fimmu.2022.1021370)
Supplement: Supplementary file 9 [file Table_1.docx]

**Supplementary Table 1.** Overview of sex distribution for R848 cohort animals.

| R848 Cohort | Cre- | | Cre+ | |
| --- | --- | --- | --- | --- |
|  | Untreated | Treated | Untreated | Treated |
| Female | 4 | 2 | 3 | 2 |
| Male | 3 | 4 | 5 | 6 |
