# Supplementary material for: The extrafollicular response is sufficient to drive initiation of autoimmunity and early disease hallmarks of lupus
Source: Front Immunol. 2022 Dec 14;13:1021370. doi: 10.3389/fimmu.2022.1021370 (PMC9795406; doi:10.3389/fimmu.2022.1021370)
Supplement: Supplementary file 10 [file Table_2.docx]

**Supplementary Table 2.** Overview of sex distribution for 564-Aicda-Cre Bcl6 cohort animals.

| R848 Cohort | Adolescence | | Adult | |
| --- | --- | --- | --- | --- |
|  | Cre- | Cre+ | Cre- | Cre+ |
| Female | 5 | 3 | 6 | 1 |
| Male | 6 | 5 | 6 | 9 |
